# Sustainable development goals (SDGs) and resilient healthcare systems: Addressing medicine and public health challenges in conflict zones

**DOI:** 10.1097/MD.0000000000041535

**Published:** 2025-02-14

**Authors:** Chinyere N. Ugwu, Okechukwu Paul-Chima Ugwu, Esther Ugo Alum, Val Hyginus Udoka Eze, Mariam Basajja, Jovita Nnenna Ugwu, Fabian C. Ogenyi, Regina Idu Ejemot-Nwadiaro, Michael Ben Okon, Simeon Ikechukwu Egba, Daniel Ejim Uti

**Affiliations:** a Department of Publication and Extension, Kampala International University, Kampala, Uganda; b Health Care and Data Management, Leiden University, Leiden, Netherlands; c Department of Public Health, School of Allied Health Sciences, Kampala International University, Kampala, Uganda; d Directorate of Research, Innovation, Consultancy and Extension (RICE), Kampala International University, Kampala, Uganda.

**Keywords:** conflict-affected areas, healthcare systems, sustainable development goals, telemedicine and mobile health clinics

## Abstract

This review explores the integration of sustainable development goals (SDGs) into healthcare organizations in conflict zones, with a specific focus on emergent models aimed at improving population health. The primary objective is to examine how innovative approaches such as telemedicine, mobile health clinics, and community health worker initiatives can be aligned with SDG targets, thereby enhancing healthcare outcomes in conflict-affected regions. The review focuses on the important areas of concern which encompasses technology, infrastructure, community engagement, and social/psychological factors. Particularly, strategies of improving existing technologies such as electronic health records and mobile health applications, enhancing health systems and community-based interventions, and integrating of mental health services are highlighted. Other focal points include advancing better water, sanitation and hygienic practices, sustainable water resource management, and other alternative financing mechanisms, such as public–private partnerships. Integrating these strategies are closely linked with the active participation of international, local governments, and affected communities in their effective implementation. This review highlights the need for monitoring and evaluation to assess intervention effectiveness and advocacy efforts to ensure that interventions supported and advocated by the international community are creating successful outcomes towards the SDG goals and improving population health in post conflict settings.

## 1. Introduction

The sustainable development goals (SDGs) and resilient health care systems’ crossroads are particularly important for responding to the multidimensional problems of medicine and public health that arise in the context of armed conflicts.^[1]^ Crises reinforce the challenges in the health sector, impede the availability of health care, and roll back the progress of (SDG 3 and SDG 16).^[2]^ This calls for the development of comprehensive strategies that address the challenges and are in harmony with the SGDs; the development of sustainable and robust health systems; and the provision of creative solutions to the problems that arise.^[3,4]^ According to the studies done, while the initial reaction to the healthcare crises in the conflict areas has improved immensely, there is still a lack of consideration of the SDG goals in these responses.^[5]^ A clear understanding of the health needs and the linkages of these needs to specific SDGs is therefore essential in order to come up with strategies that will enable the integration of these into health interventions and policies in the pursuit of achieving their goals.^[6]^ It is not only a matter of reinforcing structures but also of identifying the best practices and practices that have been effective in one context or another and can be transferred and implemented in others.^[7]^ New strategies like telemedicine and mobile health clinics can also be used to address the problems of access to and provision of healthcare services.^[8]^ Also, the capacity of building health care facilities and community-based health programs is crucial for continuing health care services during times of conflict.^[9]^ Another important area in these settings is the efficient and sustainable management of health care resources. Such strategies have to be designed to ensure effective utilization of resources in the face of constraints and disruptions and include the examination of the sustainability of practices in healthcare and their delivery and results.^[10,11]^ The SDGs give priority to the health and well-being of people, especially in areas affected by conflict and epidemics where healthcare is a luxury.^[1]^ The SDGs 3 (Good Health and Well-Being) and 16 (Peace, Justice, and Strong Institutions) point at the requirement of healthcare systems to be sustainable in such environments and able to cope with the issues arising from the conflict.^[2,3]^ While much progress has been made in identifying and meeting the basic needs of the conflict-affected communities, the analysis of the resilience-based interventions, their incorporation into the healthcare systems, and their compatibility with SDGs^[4,5]^ is limited. Most of the existing literature lacks analysis of the sustainable strategies that are important for the development of resilient health systems that can function even under the conditions of protracted crises.^[12]^ Definitely, there is a lack of literature on how best to integrate the SDG principles into the health care systems in conflict-affected areas, especially in the development of sustainable health care systems that not only meet current needs but also foster the achievement of health SDG.^[13]^ Also, there are limitations in assessing the effectiveness of new models in the continuation of healthcare and their association with other goals of the SDG.^[14]^ It is therefore important to investigate how healthcare systems that are resistant to any form of disaster can be fashioned out and put in place to cope with the 2-fold task of responding to disasters and achieving long-term goals.^[15]^ It entails evaluating the extent to which practices are in tune with SDG and coming up with new ways of delivering healthcare in conflict-affected areas that are both efficient and sustainable.^[16]^ This research aims at filling the gap within the ESRC-funded program on emergency health care and sustainable development, providing useful insights into how the 2 can co-exist with a view to improving the overall health systems in the event of conflicts.

## 2. Methodology

### 2.1. Literature search

The consolidated search for the articles was done using PubMed database, Scopus, JSTOR, EBSCOhost, ProQuest, Google Scholar, and Web of Science search engine. Some of the important terms searched for were “Sustainable Development Goals,” “healthcare system,” “conflict areas,” “telemedicine,” “mobile health clinic,” “Community Health Workers”, and “building capacity, SDG.” Only the works of the period from 2010 to 2024 were used in the insistence on relevance and the provision of updated information.

### 2.2. Inclusion criteria

Researches of health care services confined only to the context of conflict zones.

Studies conducted using the framework to advance the implementation of SDGs in healthcare organizations. Articles that highlight on such strategies as telemedicine strategy, strategic mobile clinics, as well as electronic health records (EHRs). Articles on creating, enhancing and supporting local health care systems, infrastructure, and policies in conflict-affected contexts.

### 2.3. Exclusion criteria

Only those studies which were not related to healthcare or SDGs in conflict-afflicted areas were excluded. Also, only articles written in English language were selected in order to be easily understandable. Studies that were not related to the healthcare matters or the strategies related to it were also excluded.

### 2.4. Data extraction and synthesis

Some of the major themes that were identified include: new and creative health strategies, strengthening institutions, technology, community involvement, and policies. Each study was assessed as to how well it addressed the themes that were derived from the literature namely effectiveness, challenges, and linkage with SDGs. The study conclusions were then drawn in order to understand how different strategic approaches are related to SDG principles and their effects on health care systems in conflict areas.

### 2.5. Key themes in the linkage between SDGs 3 and 16 in conflict-affected regions

A literature search to select important studies and reports was conducted and the themes developed from the research include healthcare system resilience, governance and mental health, and conflict-affected regions. These themes were health system response to conflict situations, health outcomes in conflict settings, governments and SDGs in fragile and conflict-affected countries, mental health and psychosocial support (MHPSS) in health interventions, and health as a form of conflict transformation. Theoretical frameworks were also employed in the organization of the findings. Systems thinking was especially effective in capturing the health, governance, and social systems in conflicts: multi-faceted factors influencing health and SDG achievements, including political instability, displacement, and economic stress. This framework also assisted in identifying that health systems in conflict-affected areas are not offline from other social, economic, and political systems that need to be considered for SDG achievement. The analysis was underpinned by systems thinking which views the world as a complex web of relationships, feedback loops, adaptation, and coping and the ability of health systems to learn from experience and recover from challenges posed by conflict, natural disasters or political instability. This approach also helped to avoid the situation whereby the findings are anchored on empirical evidence but have not been underpinned by a sound theoretical framework to support the analysis.

### 2.6. Aligning healthcare interventions with SDG targets in conflict zones

The integration of healthcare interventions to the SDG targets in conflict-affected areas will therefore go a long way in enhancing the health of the people and also promote stability in the affected regions.^[2,17]^ This alignment needs the identification of the population needs, the development of strategies, and the incorporation of the SDG goals in the health interventions and policy (Table 1). In order to map the health needs and focus in the conflict-affected areas to the SDG goals one has to first determine the health problems in the conflict-affected areas.^[18,19]^ In the identified settings, SDG 3 on good health and well-being for all is a key target. These comprise maternal and child health care, vaccination and treatment of communicable and noncommunicable diseases. Also, people’s mental health is an essential factor that requires attention as increased numbers of people face stress and trauma in the conflict-affected areas. Water, sanitation, and hygiene (WASH) is also crucial in avoiding disease outbreak since adequate WASH is not available in some parts of the world.^[7]^ Another goal that should also be mentioned is SDG 16 which is linked to peace, justice, and institutions. Healthcare workers and facilities should be shielded from violence and their safety should be safeguarded in order to preserve and promote healthcare.^[20]^ Defining legal and policy rules which should regulate healthcare access and provision in times of armed conflict will contribute to its preservation. Involving the communities and working with them so that they trust the health interventions and participate in the decision making process can be very helpful in increasing the usefulness and efficacy of the interventions.^[21]^ Here are some of the ways through which SDG goals can be incorporated into humanitarian health programs and policies: this means that the first thing that must be done is to undertake comprehensive health needs assessments, which will enable the identification of priorities and therefore the most pressing and important health needs of the population that needs to be addressed.^[22]^ This process assists in the improvement of targeting interventions and their implementation. Cooperation and networks are at the core of this approach. Through partnership with international bodies, state and non-state actors, nongovernmental organizations (NGOs) and community based organizations, solutions can be found to health problems. All the social determinants of health, including education, food, and economic stability, are interrelated and all affect health.^[23]^ Another important approach is the capacity building. By training and mobilizing local health workers, the provision of healthcare services can be made to be sustainable in the long run. Another distinctive of effective healthcare systems is the need of investing in the health sector infrastructure and systems at the local level to build a sustainable health care system that can cope with the impacts of conflict. Monitoring and evaluation (M&E) help in ensuring that healthcare interventions are in congruence with the SDG goals. The establishment of effective monitoring systems that enable tracking progress enhances the ability of making decisions based on the data and adjusting the interventions in accordance with them.^[24]^ It is because of that the system of constant monitoring is vital for identifying strengths, weaknesses, opportunities, and threats in a process and for making sure that the program is on the right track to meet the intended objectives. These are the roles of advocacy and awareness in this alignment process. It may be useful to encourage the integration of SDG goals in humanitarian assistance and responses at the national and international level in order to change policy and gain more support for such endeavors.^[25]^ Educating the stakeholders and the general population is a way of ensuring that they understand the significance of integrating the health care interventions with the SDG goals to ensure that they get support and funding. There is therefore the need to find ways of improving healthcare delivery in conflict zones and innovation and technology can play a huge role in this. Telemedicine, mobile health applications, and EHRs can make it easier for different patients to have access to health care, help in fastening the processes and ensure the best health outcomes.^[26]^ As the result of the proposed approach to the design of healthcare solutions that can be applied in the context of conflict zones may be more effective and sustainable.^[27]^ Therefore, linking up the healthcare interventions with the SDG targets in conflict-affected areas may be done in a manner that ensures that the basic healthcare needs of the affected populations are met while at the same time aiming at building strong institutions towards the achievement of peace and justice.^[28]^ Through the incorporation of the SDG principles into the humanitarian health programs and policies, and through the use of partnerships, capacity development, monitoring, advocacy and innovations, there is the possibility of improving on the health of populations in these high risk settings.

**Table 1:** Alignment of healthcare interventions with sustainable development goal (SDG) targets in conflict zones.

Component	Description	Relevant SDG targets	References
Identification of Healthcare Needs	Identify critical health issues in conflict zones, such as maternal and child health, vaccination, and treatment for diseases.	SDG 3 (Good Health and Well-Being)	^[2,17–19]^
Mental Health Support	Provide mental health support due to high levels of trauma and stress experienced by conflict-affected populations.	SDG 3 (Good Health and Well-Being)	^[1,2]^
WASH (Water, Sanitation, Hygiene)	Ensure access to clean water, sanitation, and hygiene to prevent disease outbreaks.	SDG 3 (Good Health and Well-Being), SDG 6 (Clean Water and Sanitation)	^[7]^
Protection of Healthcare Workers	Safeguard healthcare workers and facilities from violence to maintain service delivery.	SDG 16 (Peace, Justice, and Strong Institutions)	^[20]^
Legal and Policy Frameworks	Establish frameworks that protect healthcare access and delivery during conflicts.	SDG 16 (Peace, Justice, and Strong Institutions)	^[20]^
Community Engagement	Engage with local communities to build trust and involve them in decision-making processes.	SDG 3 (Good Health and Well-Being), SDG 16 (Peace, Justice, and Strong Institutions)	^[21]^
Needs Assessment	Conduct comprehensive needs assessments to identify and prioritize urgent healthcare challenges.	SDG 3 (Good Health and Well-Being)	^[22]^
Partnerships and Collaboration	Collaborate with international organizations, local governments, NGOs, and community groups to address complex health issues.	SDG 17 (Partnerships for the Goals)	^[23]^
Multi-Sectoral Collaboration	Address social determinants of health, such as education, food security, and economic stability.	SDG 1 (No Poverty), SDG 2 (Zero Hunger), SDG 4 (Quality Education), SDG 8 (Decent Work and Economic Growth)	^[23]^
Capacity Building	Invest in training and empowering local healthcare workers and developing local healthcare infrastructure and systems.	SDG 3 (Good Health and Well-Being)	^[23]^
Monitoring and Evaluation	Implement frameworks to track progress, enabling data-driven decision-making and continuous improvement of interventions.	SDG 3 (Good Health and Well-Being)	^[24]^
Advocacy and Awareness	Promote the integration of SDG principles in humanitarian responses and raise awareness to garner support and funding.	SDG 3 (Good Health and Well-Being), SDG 16 (Peace, Justice, and Strong Institutions)	^[25]^
Innovation and Technology	Use telemedicine, mobile health applications, and electronic health records to enhance healthcare delivery in conflict zones.	SDG 9 (Industry, Innovation, and Infrastructure)	^[26,27]^
Sustainable and Long-Term Impact	Ensure that healthcare interventions contribute to long-term stability, peace, and development in conflict zones.	SDG 3 (Good Health and Well-Being), SDG 16 (Peace, Justice, and Strong Institutions)	^[28]^

### 2.7. Mapping healthcare needs and priorities to relevant SDG targets SDG 3: Good Health and Well-being

Referring health care needs and priorities to SDG targets mainly to SDG 3 (Good Health and Well-being) and SDG 16 (Peace, Justice, and Strong Institutions) requires understanding the conflicts in the context of population needs and addressing them strategically (Table 2). The third SDG is to “ensure healthy lives and promote well-being for all at all ages.” In conflict afflicted areas, this starts with the provision of essential primary health care which entails maternal and child health.^[29,30]^ This is important since conflict greatly raises the chances of maternal and infant death. Vaccination campaigns should not be neglected as the outbreak of the preventable diseases is relatively high in the conflict-affected and congested regions. Also, both communicable and noncommunicable diseases treatment has to be easily accessible.^[31]^ Malaria, tuberculosis, HIV/AIDS, and other diseases can be aggravated by conflict, and the management of chronic diseases like diabetes and heart diseases is also poor in such situations.^[32]^ Finding solution to mental health is another significant component of SDG 3 in conflicted areas.^[33]^ The effects of psychological trauma of war and displacement will be high and may result to high rates of trauma, anxiety, depression, and other stress related disorders.^[34]^ The need for MHPSS can be critical in assisting those that are affected in order to get back on their feet and rebuild their lives.^[35]^ Combating malnutrition is important in such settings. Conflict also tends to affect food supply chains, and this implies that there will be sparse food production, and hence food insecurity and malnutrition. Preventative measures such as nutrition relief feedings, nutrient supplements, and health education to prevent cases of malnutrition are crucial in order to attend to the needs of the needy and most especially the children.^[36]^ WASH are basic necessities that help in avoiding diseases in conflict areas. This is to mean that availability of water, sanitation facilities, and hygiene products are very crucial in enhancing health standards among the populace.^[37]^ The worst WASH situation may result in the outbreak of water-related diseases such as cholera and dysentery which are very fatal especially among the affected vulnerable groups. SDG 16 is on peace, justice and institutions; it states that.^[38]^ This involves the safety of the health care personnel and structures within areas of armed conflict. The care givers are often vulnerable in conflict areas and hence their protection is important to guarantee the continuity of quality health care services.^[39]^ It is for this reason that advocacy for these workers and institutions to be safe from violence must be pursued. Also, ensuring legal and policy-based measures that support health care accessibility and delivery in the context of conflict is another dimension of SDG 16.^[7]^ These frameworks can help to define the necessary shape and direction of healthcare interventions so that they are both feasible and effective.^[40]^ Healthcare interventions in conflict-affected regions relies on community participation and trust. Involvement of the local communities in the decision making process is important because it makes the interventions appropriate to the culture of the local people and hence more likely to be accepted by the community.^[41]^ Lack of trust between the healthcare providers and the communities can hinder the health providers from getting the desired cooperation and participation from the communities in the health programs and this could affect the health of the people.^[42]^ This paper shows that to link healthcare needs and priorities to the SDG targets in conflict zones, a more elaborate approach must be used so that while attending to existing health challenges, efforts are also made towards creating the conditions for stability and development.^[43]^ Essentially through increasing basic healthcare services, mental health, nutrition, WASH, protection of healthcare workers and facilities, legal and policy improvements and community involvement, a functional and efficient and more importantly sustainable health system that supports the objectives of both SDG 3 and SDG 16 can be realized.

**Table 2:** Mapping of healthcare needs and priorities to relevant SDG targets, particularly SDG 3 and SDG 16.

Healthcare need/priority	Description	Relevant SDG targets	References
Maternal and Child Healthcare	Ensure access to maternal and child healthcare to reduce risks of maternal and infant mortality, especially in conflict zones.	SDG 3 (Good Health and Well-being)	^[29,30]^
Vaccination Programs	Prioritize vaccination to prevent outbreaks of vaccine-preventable diseases in conflict areas.	SDG 3 (Good Health and Well-being)	^[31]^
Treatment for Diseases	Provide treatment for communicable diseases (e.g., malaria, TB, HIV) and manage noncommunicable diseases (e.g., diabetes, heart disease).	SDG 3 (Good Health and Well-being)	^[32]^
Mental Health Support	Offer mental health and psychosocial support to address trauma, anxiety, depression, and other stress-related disorders in conflict-affected areas.	SDG 3 (Good Health and Well-being)	^[33–35]^
Addressing Malnutrition	Implement emergency feeding programs, nutritional supplements, and education to combat malnutrition and ensure adequate nutrition for vulnerable groups.	SDG 3 (Good Health and Well-being)	^[36]^
Water, Sanitation, and Hygiene (WASH)	Ensure access to clean water, sanitation facilities, and hygiene products to prevent waterborne diseases and maintain public health.	SDG 3 (Good Health and Well-being), SDG 6 (Clean Water and Sanitation)	^[37]^
Protection of Healthcare Workers	Safeguard healthcare workers and facilities from violence to maintain uninterrupted healthcare services.	SDG 16 (Peace, Justice, and Strong Institutions)	^[38,39]^
Legal and Policy Frameworks	Establish and enforce legal and policy frameworks that protect healthcare access and delivery in conflict zones.	SDG 16 (Peace, Justice, and Strong Institutions)	^[7,40]^
Community Engagement and Trust-Building	Engage local communities in decision-making to ensure culturally appropriate interventions, improve cooperation, and build trust between providers and communities.	SDG 3 (Good Health and Well-being), SDG 16 (Peace, Justice, and Strong Institutions)	^[41,42]^
Comprehensive Approach	Address immediate health needs while promoting long-term stability and development through a focus on key healthcare priorities.	SDG 3 (Good Health and Well-being), SDG 16 (Peace, Justice, and Strong Institutions)	^[43]^

SDG = sustainable development goal.

### 2.8. Strategies for integrating SDG principles into humanitarian health programs and policies

The incorporation of SDG principles to the existing humanitarian health programs and policies is a process that calls for a strategic planning to tackle the numerous and changing challenges of the conflict-affected areas (Table 3). The first of these is the so-called needs assessment in which an attempt is made to determine the most important healthcare problems.^[44]^ These should be comprehensive and should include data gathering and analysis in order to identify the health status of the population, the resources, and the health system.^[45]^ By mapping out the gaps and choosing the interventions that are in line with the SDG targets, health and human performance can work on the areas which have the biggest potential to produce results and therefore spend the limited resources wisely. These initiatives require partnerships and collaborations as highlighted in the following.^[46]^ This way, the cooperation with international organizations, local authorities, NGOs, and communities can be used to mobilize many-sided support and experience.^[47]^ This strategy can help in sharing of priceless information, expertise, and capital that are scarce assets where implementation of strategies takes place. It is important to work together with other sectors to deal with social determinants of health including education, food security, and economic status.^[48]^ All these determinants are closely related to health status and their improvement calls for intersectoral action. The third approach that can be utilize in the integration of SDG principles into humanitarian health programs is through capacity building.^[49]^ Therefore, investing in the further education of the local healthcare workers will help the continued provision of healthcare in the long-run. Supporting the development of the local health infrastructure and systems that will enable a health system to recover and function effectively in the face of conflict is also important.^[38,50]^ This involves constructing of hospital, clinics and other health facilities, and also the provision of medical supplies and equipment. M&E are essential in the achievement and impact of health interventions and their relevance to the SDG targets 5. The development of proper M&E structures make it easier to track the progress that has been made and the places where things are going wrong. Having evidence and data as a foundation for decision making in policy enables policies and interventions to be made in light of best knowledge and be flexible enough to be changed when other information is received.^[51]^ This approach requires advocacy and awareness therefore these 2 aspects are fundamental in this approach. Promoting the consideration of SDGs in humanitarian actions on the national and international levels can contribute to policy shifts and enhance stake for these actions.^[52]^ To increase stakeholder and public understanding of the significance of ensuring that healthcare activities relate to SDG objectives, it is possible to foster a more significant level of backing and financing. This advocacy can also support the understanding of the linkage between health and development, and therefore, the need for action on health is as important as taking action on development.^[53]^ Innovation in technology in the health sector has a great potential of enhancing the effectiveness of the health care delivery even in the conflict areas.^[54]^ Some of the telemedicine, mobile health application, and EHRs can help improve the quality of health care services delivery.^[55]^ Some of the examples of the use of telemedicine include remote consultations and support to healthcare workers in the field; while mobile health applications can be used in the promotion of health education and monitoring.^[56]^ Adaptable and flexible healthcare solutions have to be developed and deployed that can be customized to the conflict environment.^[57]^ These solutions have to be adaptable and easily scalable in order to address the dynamic requirements and environment of the people. Hence, the implementation of SDG principles in humanitarian health programs and policies can be done through a number of steps, such as needs assessment and identification, partnerships and networking, capacity development, M&E of achievement, advocacy and raising awareness, and the application of innovation and technology.^[58]^ Thus, it will be possible to design better and more efficient health care interventions that can meet not only the current health requirements but also the long-term development needs.^[59]^

**Table 3:** Strategies for integrating sustainable development goal (SDG) principles into humanitarian health programs and policies.

Strategy	Description	Relevant SDG targets	References
Comprehensive Needs Assessments	Conduct thorough needs assessments to identify healthcare challenges, resource availability, and infrastructure gaps in conflict zones.	SDG 3 (Good Health and Well-being), SDG 17 (Partnerships for the Goals)	^[44,45]^
Partnerships and Collaboration	Foster partnerships with international organizations, governments, NGOs, and community groups to leverage resources, expertise, and knowledge.	SDG 17 (Partnerships for the Goals)	^[46,47]^
Multi-Sectoral Collaboration	Address social determinants of health, such as education, food security, and economic stability, through coordinated multi-sectoral efforts.	SDG 1 (No Poverty), SDG 2 (Zero Hunger), SDG 4 (Quality Education), SDG 8 (Decent Work and Economic Growth)	^[48]^
Capacity Building	Invest in training local healthcare workers and developing healthcare infrastructure to ensure long-term sustainability and resilience.	SDG 3 (Good Health and Well-being), SDG 9 (Industry, Innovation, and Infrastructure)	^[38,49]^
Monitoring and Evaluation	Implement robust monitoring and evaluation frameworks to track progress, inform policy decisions, and adapt interventions as needed.	SDG 3 (Good Health and Well-being), SDG 16 (Peace, Justice, and Strong Institutions)	^[50,51]^
Advocacy and Awareness	Advocate for the integration of SDG principles at national and international levels to drive policy changes and raise awareness among stakeholders.	SDG 3 (Good Health and Well-being), SDG 17 (Partnerships for the Goals)	^[52,53]^
Innovation and Technology	Utilize innovative technologies, such as telemedicine and mobile health apps, to improve healthcare delivery and access in conflict zones.	SDG 3 (Good Health and Well-being), SDG 9 (Industry, Innovation, and Infrastructure)	^[54–57]^
Adaptable Healthcare Solutions	Develop and deploy flexible, scalable healthcare solutions tailored to the unique challenges of conflict zones.	SDG 3 (Good Health and Well-being)	^[57]^
Comprehensive Approach	Integrate these strategies to create more effective and sustainable healthcare interventions that contribute to both immediate and long-term goals.	SDG 3 (Good Health and Well-being), SDG 16 (Peace, Justice, and Strong Institutions)	^[58,59]^

### 2.9. Building resilient healthcare systems in war-torn areas

**Figure 1.**
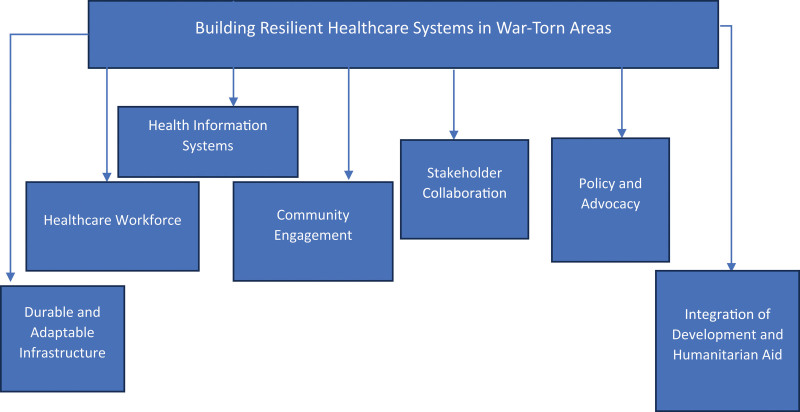
Strategies involved in building resilient healthcare systems in war-torn area.

Strengthening health care systems in war zones is quite a challenging yet critical process which is required to guarantee that people in such conflict regions are able to get the health care they require (Fig. 1). Improving the healthcare sectors and structures in such situations calls for coordinated intervention aimed at the present whilst at the same time preparing for the future.^[60,61]^ Therefore, one of the strategies that can be used in order to enhance the capacity of health care systems is to address the issue of infrastructure and particularly the physical infrastructure. This entails creating and sustaining structures and equipment in health facilities that are constructed in a manner that can withstand the effects of war and are structures that can be easily transportable as the need arises.^[62]^ These facilities should be provided with some basic medical items and equipment and this can be a major task given the current supply chain disruptions. Ensuring that supply lines are safe and accumulated supplies are well positioned can go a long way in combating these difficulties. Another important factor is that there should be adequate and competent health workforce.^[63]^ Hospitals and clinics in conflict areas are usually devoid of enough doctors and other health professionals because most of them have been either killed or have fled the region. Efforts to educate the local health personnel are important because they will be in the community.^[64]^ The curricula of the training programs should include not only the clinical aspects but also the aspects related to the emergency and traumatic care that is especially important in the context of conflicts. Further, managing mental health of the healthcare workers can assist them in dealing with stress while practicing in such conditions.^[65]^ Another important approach is the promotion of effective health information systems. Such parameters as accuracy and timeliness of the data collection can enhance the organization of health care services, increase the efficiency of their usage, and also help in combating diseases.^[66]^ In the conflict areas, it is hard to implement the usual data collection process, and the use of technology in form of mobile health applications, and remote monitoring systems can therefore be useful.^[67]^ Some of these technologies can help in the exchange of information concerning the health facilities, to map out the spread of disease and manage patients’ status in regions with poor infrastructure. Healthcare organizations also need to engage the community as well as get their participation in the development of strong and sustainable healthcare systems.^[68]^ Community participation in the design and delivery of health interventions compliments the requirements of the community and the cultures that surround it. Community health workers (CHWs), in this case, can help in the prevention of such a situation through being the link between the healthcare system and the community.^[69]^ They can educate people on how to maintain health, assess and report on the health concerns, and promote and participate in vaccination, and other health campaigns. Improvement of healthcare systems in conflict areas also depends on the availability of effective cooperation with different partners. It means that international organizations, NGOs, local governments and communities has to combine their resources and knowledge.^[70,71]^ This cooperation can assist in the consideration of the health problems in conflict areas as complex and including not only the prevention and treatment of diseases, but also the social issues such as access to the food, shelter, and education. The third approach is the creation of policies that help to safeguard health care services in the course of conflict. National and international advocacy can be useful in making sure that healthcare facilities and personnel are protected by international humanitarian law.^[72]^ More should be done to ensure that those who perpetrate these violations are brought to book so that the violence against the health care systems and personnel is contained. Lastly, promoting resilience in healthcare systems means, inter alia, paying attention to the capacity building and development in addition to the emergency response.^[73]^ This entails ensuring the provision of assistance in a way that is in harmony with the development objectives of constructing robust health systems. For example, it is possible to invest in education and training of the future healthcare personnel, or construction of water and sanitation facilities that would positively affect people’s health even after the conflict is over.^[74]^ Strengthening of healthcare systems in conflict-affected regions has to be a holistic process to meet the current requirements as well as prepare for the future. Thus, through constructing resilient and sustainable structures, building the capacity of local health workforce, using ICT in health systems, involving communities, promoting partnerships, campaigning for the preservation of health services, and integrating development and humanitarian assistance it is possible to build health systems that are able to operate in contexts of crisis and continue delivering basic services to the affected populations.^[75]^

### 2.10. Lessons learned from successful resilient healthcare models in conflict zones

**Figure 2.**
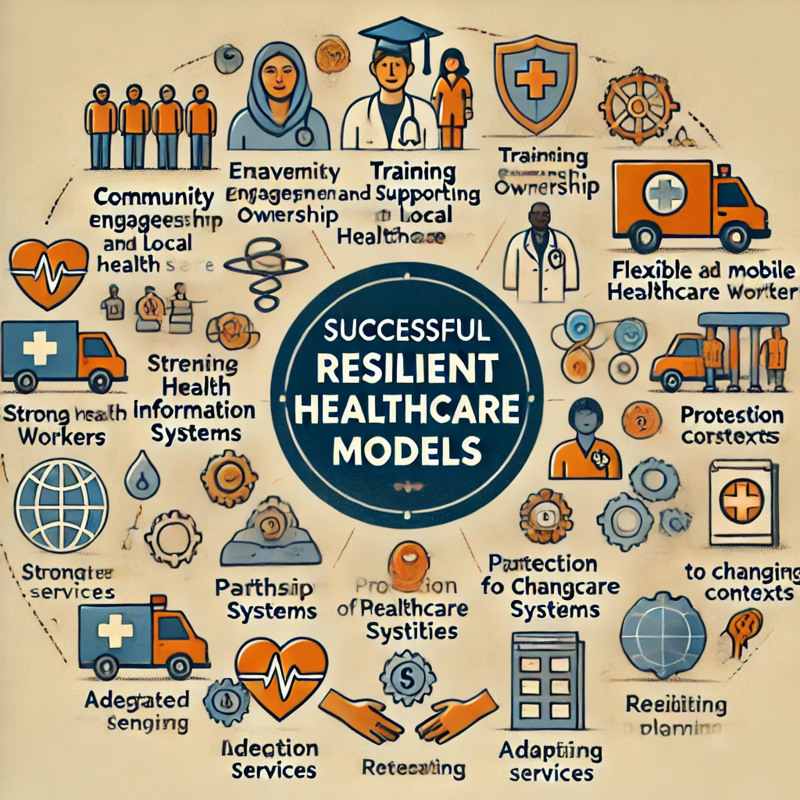
Lessons learned from successful resilient healthcare models in conflict zones.

The examples of the effective Rural Health Clinics in the conflict-affected areas can be used in other settings with similar challenges that are also experiencing similar conditions.^[76]^ These models as depicted in Figure 2 shows how strategic planning, community involvement, and innovations are able to establish strong health care systems that can function effectively in any given circumstances.^[77]^ Some of the experience of using such approaches can be seen from Table 4.

**Table 4:** Lessons learned from successful resilient healthcare models in conflict zones.

Lesson	Description	Examples	References
Community Engagement and Ownership	Involves local communities in planning and implementing health services, fostering trust, and ensuring culturally appropriate care.	Syria: Community Health Workers (CHWs) delivering essential health services.	^[78,79]^
Training and Supporting Local Healthcare Workers	Investing in training programs for local healthcare workers to maintain services in challenging conditions.	Afghanistan: Training midwives and nurses for maternal and child health.	^[80,81]^
Flexible and Mobile Healthcare Delivery	Utilizing mobile clinics and telemedicine to reach remote or dangerous areas, ensuring continuity of care.	Yemen: Mobile clinics providing essential medical services in remote areas.	^[82,83]^
Strong Health Information Systems	Accurate data collection using mHealth technologies for efficient healthcare delivery and outbreak tracking.	DRC: mHealth tools for disease tracking and patient management.	^[84,85]^
Partnerships and Collaboration	Building partnerships with international organizations, NGOs, and community groups to enhance healthcare delivery capacity.	South Sudan: Ministry of Health collaborating with NGOs and community organizations.	^[86,87]^
Protection of Healthcare Workers and Facilities	Advocating for the safety of healthcare workers and facilities to reduce attacks and ensure service continuity.	Somalia: Advocacy and initiatives for safer healthcare environments.	^[88,89]^
Integrated Services	Combining health services with other humanitarian efforts like nutrition, WASH, and mental health for better outcomes.	Iraq: Integrated health programs addressing multiple needs.	^[90,91]^
Sustainable Funding and Resource Allocation	Securing diverse funding sources and managing resources transparently to build trust and ensure effectiveness.	Various: International aid, local government support, and private donations.	^[92,93]^
Adapting to Changing Contexts	Continuously adapting strategies to changing conditions, such as new refugee influxes or disease outbreaks.	Lebanon: Adapting healthcare strategies for Syrian refugees	^[94,95]^
Resilience and Long-term Planning	Focusing on building the capacity of local health systems for long-term recovery and strengthening.	Liberia: Post-conflict health system strengthening initiatives.	^[96,97]^

### 2.11. Community involvement and participation

The most notable of these is the significance of involving the community as a key lesson. Effective models of health care engage the communities in the development and delivery of the health care services.^[78]^ For instance, in Syria, CHWs have been empowered to provide health care services, health promotion and other health related information, and act as bridges between the communities and the formal health care systems. This approach building trust and guarantees that the health services will be culturally sensitive and will correspond to the real needs of the people.^[79]^

#### 2.11.1. Capacity building and support of local health care givers

**Figure 3.**
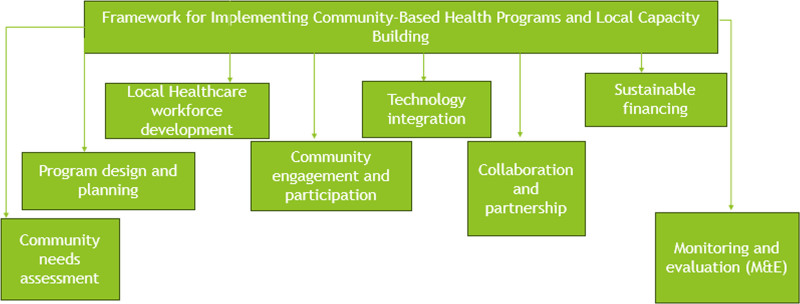
Structured overview of the essential components and steps involved in implementing community health-based programs and building local capacity.

It is crucial that more attention should be paid to the training and development of local health personnel as shown in Figure 3. For instance in Afghanistan, because of the conflict there, training of local midwives and nurses has been crucial in the provision of maternal and child health services.^[80]^ These programs not only equip them with the required knowledge and skills but also see to it that the health care providers are from the community and thus have high propensity of staying on even in the most difficult circumstances.^[81]^

#### 2.11.2. Innovative and easily transportable health care services

Reliability of healthcare delivery is essential. Mobile clinics and telemedicine have been seen to be useful in conflict areas where there are few or no fixed structures which can be used for health care.^[82]^ Mobile clinics have been used in Yemen to ensure that health care is taken to the people in the most unreachable and volatile regions, hence minimizing the risks for both the patients and the health care givers.^[83]^

#### 2.11.3. Health information systems that are strong

Healthcare data collection is crucial in conflicts, and therefore, data should be collected accurately and on time. mHealth technologies have been effective in many conflict-affected areas 88. For example, in the Democratic Republic of Congo, the mHealth applications have been deployed in tracking diseases incidence, controlling patients’ data, and planning for health services, through this making health care more effective and timely.^[85]^

#### 2.11.4. Partnerships and collaboration

It is also a characteristic of effective humanitarian and community models in conflict-affected regions that the partnerships are formed with international (I) and NGOs, local governments, and community groups.^[86]^ In South Sudan, the collaboration between the Ministry of Health, international NGOs, and other community based organizations have encouraged resource and experience sharing to improve on the ability to provide health care services in the face of the existing challenges.^[87]^

#### 2.11.5. Protection of HCW and healthcare facilities

Taking care of the safety and security of the health care workers and facilities is paramount. In conflict-affected areas, the need to ensure that health facilities are protected in compliance with the principles of international humanitarian law can help to minimize the incidence of attack on the health care delivery system.^[88]^ For instance, in Somalia local and international efforts have been made to ensure that there are proper measures that have been put in place to protect health care givers and clients.^[89]^

#### 2.11.6. Integrated services

Coordinating health services with other forms of assistance has been successful. In Iraq, health programs that include nutrition, WASH, and mental health have been found to have better performance.^[90]^ This approach recognizes the fact that health is not confined to the individual and that social determinants of health must be tackled in conflict-affected environments.^[91]^

#### 2.11.7. Resource endowment and sustainability: how best to support them?

Sustainable funding is important as is the management of resources. Many of the effective programs have had multiple sources of funding such as foreign grants, government funding, and private contributions.^[92]^ In this manner, the management of these resources is clear and understandable and it is easy to determine whether the funds are being spent properly.^[93]^

#### 2.11.8. Contextualization

Flexibility is crucial as- and when environments fluctuate in a short span of time. In Lebanon for instance, the healthcare programs for the Syrian refugees have had to alter their approaches in line with the dynamics of the situation on the ground including the arrival of new batches of refugees or the occurrence of epidemics.^[94]^ This flexibility guarantees that the health care services remain useful and efficient.^[95]^

#### 2.11.9. Resilience and long term planning

Finally, the importance of thinking long-term and building resilience even as we meet short-term requirements cannot be overemphasized. Best practices also include practices that help to develop the local health systems’ resilience and ability to bounce back and improve in the future.^[96]^ In Liberia, there was a training of health care personnel, construction of facility, and formulation of health policies all of which enhanced the health system after conflict.^[97]^

### 2.12. Innovative approaches to medicine and public health in conflict settings

New strategies for medical practice and health care in conflict areas are crucial to the management of the problems associated with conflict (Table 5). A key advancement is telemedicine which allows for remote medical examination, diagnosis, and even follow up through the use of technology in a bid to overcome challenges that are occasioned by physical insecurity and destroyed healthcare infrastructure.^[118,119]^ Mobile clinics also have significance in that they offer mobile and flexible health care delivery to reach people in these hard to reach and insecure places to deliver basic services such as immunization, ante-natal care, and other outpatient care. Another interesting approach is community-based health programs which involve the community members in the management of their health.^[120]^ They usually entail raising awareness of local health workers who in turn can offer primary health care services and health education within the community thereby building the communities’ health care capacity and ensuring the continuation of health interventions.^[121]^ This approach is also effective in enhancing the adaptation of the communities and makes it easier to embrace the health care services provided hence making them to be acceptable in the region. Also, new approaches in supply chain management including delivery of medical supplies by drones assists in provision of regular and adequate stock of drugs and equipment in the conflict zones thus reducing risks of stock out and closure of health facilities.^[122]^ In conclusion, these innovative approaches are vital to increase the coverage, sustainability and quality of health services in conflict-affected areas thus improving health status and population’s resilience. The following are the increment in the use of telemedicine, mobile clinics, and other technologies in increasing the access to health care. New ideas in medicine and public health are therefore necessary in order to solve some of the problems associated with conflict.^[123]^ These strategies utilize technology, community, flexibility, and cross-sectoral integration to ensure that the fundamental health services are provided amid the conflict. Here are some key innovations that have proven effective: here are some key innovations that have proven effective.

**Table 5:** Innovative approaches to medicine and public health in conflict settings.

Innovation	Description	Examples	References
Telemedicine and Mobile Health (mHealth)	Enables remote consultations, diagnostics, and follow-ups, reducing the need for travel in dangerous areas.	Syria: Telemedicine platforms connecting local workers with international specialists.	^[98,99]^
Mobile Clinics	Provides flexible and mobile healthcare delivery to inaccessible or destroyed areas, offering services like vaccinations and emergency care.	Yemen: Mobile clinics reaching remote conflict-affected populations.	^[100,101]^
Drones for Medical Supply Delivery	Delivers essential medical supplies to hard-to-reach areas, overcoming terrain and access challenges.	Rwanda: Drones delivering blood supplies to remote clinics.	^[102,103]^
Community Health Worker Programs	Trains and empowers local health workers to provide basic services, education, and disease surveillance.	Liberia: Community Health Workers improving maternal and child health outcomes.	^[104,105]^
E-health and Digital Health Records	Utilizes digital systems to track patient information and improve coordination, even in displaced populations.	Conflict zones: Digital health records maintaining continuity of care.	^[106,107]^
Psychosocial Support and Mental Health Interventions	Provides mental health support through mobile units, tele-counseling, and community programs to address trauma and stress.	Gaza: Community centers offering mental health services alongside other support.	^[108,109]^
Water, Sanitation, and Hygiene (WASH) Innovations	Implements new technologies and practices to improve access to clean water and sanitation, preventing disease outbreaks.	South Sudan: Solar-powered water purification units providing clean drinking water.	^[110,111]^
Cross-Sector Collaboration	Integrates health with other sectors like nutrition and education for comprehensive interventions.	Afghanistan: Health and nutrition programs providing food aid and health services.	^[112,113]^
Rapid Response Teams	Mobilizes quickly to respond to health crises, providing immediate care and coordinating with local authorities.	Various conflict zones: Rapid response teams addressing sudden outbreaks and emergencies.	^[114,115]^
Innovative Financing Mechanisms	Uses novel financial models like microinsurance and crowdfunding to ensure sustainability of healthcare programs.	Nigeria: Microinsurance schemes enabling community-funded healthcare access.	^[116,117]^

#### 2.12.1. Telemedicine and mobile health also commonly referred to as mHealth

Technologies such as telemedicine and mHealth have brought a lot of change in the provision of health care in the conflict regions. These technologies include telemedicine, which allows for remote consultations, diagnosis and treatment of patients thereby sparing the lives of patients, and healthcare providers from going through dangerous territories.^[98]^ For instance, the telemedicine platforms have been widely employed in Syria in order to enable local healthcare professionals to communicate with the international professionals and receive advice and consultations in real time.^[99]^ Mobile health applications can help in tracking diseases, educating people on their health, and communication between the healthcare givers and the patients thus enhancing the health care delivery systems.

#### 2.12.2. Mobile clinics

Mobile clinics are the mobile health care delivery structures in conflict zones where other structures may not be reachable or have been ruined.^[100]^ Such clinics can be easily mobilized to areas with high disease burden and provide services such as outpatient, maternal and child health, immunization and emergency treatment. In Yemen mobile clinics have been very useful in ensuring that people in hard to reach and conflict areas are reached and health care is continued despite the ongoing conflict.^[101]^

#### 2.12.3. Delivery of medical supplies via drones

Delivery of medical supplies by drones have become more common in conflict areas to reach patients in remote locations.^[102]^ These drones can fly over treacherous and undrivable terrains to make sure that the much needed medicines, vaccines, and blood products get to where they are needed. In Rwanda, drones have been employed effectively in the delivery of blood to remote health facilities thus cutting down on time and hence saving lives.^[103]^

#### 2.12.4. CHW programs

The training and mobilization of local CHWs is an excellent approach in conflict-affected region. Due to their close association with the targeted communities CHWs can offer primary healthcare, health promotion, and disease reporting.^[104]^ This is because this approach goes further in ensuring that people are able to access health care services while at the same time creating trust within the community. In Liberia, CHWs have been used to enhance the health of mothers and children in conflict prone regions.^[105]^

#### 2.12.5. Electronic health and electronic health records

Integrating electronic health systems, and EHRs increases the effectiveness and organization of the healthcare delivery.^[106]^ The digital records can help in tracking of patient’s history, treatments and progress, especially in those who are on the move or have been displaced. In conflict-affected areas where paper based records can be easily misplaced or destroyed, the use of digital health can ensure that patients’ records are easily and safely retrievable.^[107]^

#### 2.12.6. Psychosocial support and mental health promotion and prevention, treatment, and care

New models of mental health care are needed in conflict-affected areas since trauma is very common.^[108]^ Mobile health clinics, telephonic counseling, and other group counseling services can help in addressing psychosocial needs. For instance, in Gaza, the community centers provide mental health services among other social services thus providing a comprehensive care for the affected people.^[109]^

#### 2.12.7. New developments in WASH

It is, therefore, important to enhance WASH conditions in order to avoid outbreak of diseases in regions with conflict. There are examples of portable water purification systems, solar-powered sanitation where people are educated on hygiene practices within their communities and this has been seen to work well.^[110]^ In South Sudan, the solar water purification has offered hope to the displaced persons by providing them with clean water for drinking and thus reducing the cases of water-borne diseases by.^[111]^

#### 2.12.8. Cross-sector collaboration

With the interdependence between health, nutrition, education, and economic sectors, there is potential for more appropriate and impacts approaches.^[112]^ It is for this reason that integrated programs that target a number of needs at the same time will be effective in conflict areas. For instance, in Afghanistan, services that combine health and nutrition have been able to distribute food, educate people on nutrition and offer health care services to needy individuals; both for the short term and long term health needs of the affected populations.

#### 2.12.9. Rapid response teams

In conflict zones there is need to set up rapid response teams that can be deployed at short notice in case of health emergencies.^[114]^ These teams which include the health care workers, logisticians, and other support staff can be sent to areas that are affected by an outbreak or in humanitarian crises. Thus, the ability to attend to the people’s health needs as well as liaise with the various government agencies can help in minimizing the effects of health related disasters.^[115]^

#### 2.12.10. Innovative financing mechanisms

Thus, sustainable financing is critical to the availability of health care services in conflict areas. This is where new ways of funding such as microinsurance schemes, health savings accounts, and crowdfunding comes in to ensure that healthcare programs are funded.^[116]^ In conflict ridden regions of Nigeria, microinsurance has allowed communities to contribute towards a pool and receive health care even in the face of economic disturbances.^[117]^

### 2.13. Implementation of community-based health programs and local capacity building

Therefore, the design and development of community-based health programs and strengthening of local structures must involve a whole-of-community and flexible approach in order for the health care systems to be sustainable especially in areas of conflict or limited access to health care.^[6]^ The process starts with identification of the community’s needs through community assessment. This includes interacting with the opinion makers, health care providers, political leaders, and the community inhabitants in order to get input from all sides.^[8]^ This is where data collection is quite important and this can be done through surveys, focus groups, and review of previous health data.^[47]^ The following data assist in the assessment of the population’s health status, the types of diseases that are common in the community, the coverage and accessibility of health care services, and the existing health care facilities.^[85]^ This leads to a gap analysis where one is able to identify certain aspects that need to be addressed this may include maternal health, infectious disease prevention or mental health. According to the needs assessment, the following is the next step which is designing and planning the health program. The goals are well defined, measurable and respond to the stated needs while also integrating with other health and development goals such as the SDGs 6 and 7. The distribution of resources is done in a very strategic manner including finance, staff, materials, and equipment. The budget is prepared and efforts are made to secure funds from the government, international organizations, and individuals and organizations.^[87]^ This hence leads to the development of an implementation plan which includes time schedules, who does what, and the specific steps to be taken and this is developed bearing in mind that flexibility is important especially in areas of conflict. Strengthening of the local health care workforce is one of the important strategies for enhancing the health care sector. Recruitment is a process of identifying those people in the community who can be trained to be CHWs.^[63]^ This helps in promoting cultural sensitivity and gain the confidence of the society. The comprehensive training is carried out for the graduates in the skills of clinical, public health, emergency, and health education.^[119]^ To ensure that the healthcare workers are up to date with the current skills they receive both new employee training and continuing education. Other organizational structures that can be put in place to assist the healthcare workers are supervision, mentoring and peer support to enhance performance, and cope with stress at the workplace.^[74]^ It is important therefore to involve the community and have them participate in the program. Local health committees are established to include members of the community and these committees are used to oversee the implementation of the program as well as to make sure that the program reflects the community’s interest and preferences.^[47]^ While local health committees made up of various members of the community are put in place to oversee the implementation of the program and ensure that the program is relevant to the community.^[47]^ The public health promotion activities are implemented through different forms such as group discussions, radio talks, and social media forums for creating awareness on health concerns, precautionary measures, and services that are available to the public.^[99]^ Feedback from the community is received on regular basis through suggestion boxes, community meetings, and questionnaires to enhance the program. Health programs can therefore be delivered to a larger and more impactful audience through the use of technology.^[56]^ Mhealth applications are used for purposes of health promotion, appointment scheduling, disease monitoring, and data gathering especially in hard to reach or volatile regions. Telemedicine platforms are used to conduct remote consultations and get the help of specialists which are quite effective in the areas with poor access to the health care workers.^[57]^ These are created to reflect current patient information in a bid to enhance patient care especially for the nomadic or those in transitional circumstances.^[67]^

Another important factor is collaboration and partnerships for the purpose of resource and skills mobilizations. Collaborations are made with the local and national government in order to support the health polices and to make the program sustainable.^[93]^ These include offering technical assistance, financial and logistical support, and assistance in the implementation of the programs. Private sector is involved in funding, technological innovations, and supplies and aids. The issue of sustainable financing is therefore important in the sustain ability of health programs.^[63]^ There is a pursue to obtain funds from various sources such as government budgets, international donors, private sector, and community fundraising. Microinsurance products and community based financial models are proposed for community members to contribute towards a health fund for basic needs. Cost-sharing mechanisms are adopted where possible to sustain the services while at the same time making the services affordable in order to promote revenue for health services.^[85]^ There is therefore the need for continuous M&E so as to ensure that the programs are of value and applicable. There are clear markers of how the program affects health status, services and the community, and its satisfaction level.^[74]^ Data collection and analysis are done on a routine basis in order to assess performance while feedback from M&E activities is used to inform program management and alter program delivery. The successes are captured and disseminated while the failures are also recorded for future reference; good practices are expanded to other regions so that other regions can access the interventions.^[52]^ Community-Based Health Programs and Local Community-Based Programs can be defined as an articulated, systematic, and comprehensive process which aims at involving the communities, developing human recourses in the communities, utilizing technology, forming strategic alliances, ensuring sustainable funding sources, and on-going program improvement.^[97]^ This approach helps not only to solve the current problems in the healthcare sector, but also develops a system that will be able to overcome the difficulties and challenges in the future.

### 2.14. Sustainable health solutions and resource management

This is important as sustainable health solution and management of resources are key to ensuring that health care delivery is sustainable in conflict areas which are characterized by scarce resource and frequent disruption.^[124]^ Some of the strategies are: efficient utilization of resources through proper planning and scheduling, this way the essential health needs are well catered for despite the shortage of supplies. Other factors that should be considered include: building on local resources and assets; these include empowering the local health care workforce and working with local suppliers to decrease reliance on imported items.^[125]^ One of the other major factors is the integration of renewable energy solutions including solar power to support the function of health facilities even during the power outages. It is also important to have water conservation and recycling systems in place so as to ensure that there is proper hygiene and sanitation.^[126]^ Also, there are other measures that have been proposed for adoption in the healthcare sector to make it sustainable; for instance, the ways of managing medical wastes through disposal and recycling and the use of bio-degradable materials where possible.^[127]^ These practices need to be checked and assessed on a regular basis to determine their efficiency and appropriateness with a view of making necessary changes. Thus, concentrating on sustainable approaches and proper utilization of the resources, it is possible to strengthen the healthcare facilities in the conflict areas and avoid the interruption of the services provided.^[128]^

### 2.15. Strategies for managing healthcare resources sustainably amidst scarcity and disruption

This paper argues that, to meet the healthcare needs in the face of scarcity and disruption, there is the need to put in place a system that can address the current challenges while at the same time taking into consideration the future.^[102]^ In situations where availability of resources is a challenge and the environment is prone to disruptions for instance in conflict regions or areas with little or no access to healthcare, it is important to put in place proper management measures in a bid to enhance the delivery of health care services in a fair manner. Therefore, the assessment of health care resources as well as the needs that have to be met is a cornerstone of sustainable health resource management.^[103]^ This includes physical assessment of the existing stock of drugs and diagnostic equipment and personnel, and assessment of the health status of the populace. This way, through conducting the inventory and needs assessment, healthcare organizations will be able to know where there are weaknesses and allocate resources where they will be most helpful.^[104]^ This step is crucial to decision making particularly on allocation and to help prevent wasteful activities. Strategic management of resources is one of the key factors that define the effective management. According to the above, resources should be directed depending on the needs and the possible outcomes that can be expected. For instance, in special circumstances such as outbreak of disease or emergency, priority should be given to the most important sectors including: health service delivery especially in the emergency units and availability of drugs.^[105]^ This prioritization helps in the provision of services in the order of their importance while conserving the available resources. Furthermore, creation of a tiered system can assist with the utilization of resources in that it divides needs into categories based on the severity of the need and then allocates resources thus.^[106]^ Some of the ways through which the healthcare can improve its resources management is through the application of the lean management principles. This include eliminating unnecessary tasks, cutting down the unnecessary activities, and increasing productivity. As an example, through the use of inventory control systems, it is possible to monitor the utilization and availability of medical commodities in the real time to avoid stock out or over stocking.^[109]^ It also helps in improving the efficiency and reducing the variability in the resource utilization while ensuring that the care is provided in the right manner. Of special importance is the enhancement of local institutions’ ability to manage resources sustainably.^[112]^ Building the capacity of local health care workers in the management of resources; logistics, supply chain, and emergency preparedness can go a long way in building a strong health care workforce in the face of resource limitations. Furthermore, encouraging local production and procurement of medical supplies will also help to minimize on the dependence on foreign sources and thus, enhance the supply chain system.^[5]^ This can be done through promoting the production of key medical commodities within the country including drugs and testing equipment to reduce on the impact of supply chain interruptions and costs. This paper has established that cooperation and alliances are important in the sustainable management of health care resources. Therefore, engaging international organizations, NGOs, and other private actors may contribute to the process and offer their resources and experience.^[97]^ Examples of co-operation may be exchange of experiences, combining assets, and measures in relation to crisis management. For instance, collaboration with pharmaceutical firms may result in the delivery of drugs or assistance in enhancing the infrastructure of the local health care facilities.^[74]^ Other forms of innovations such as financing mechanisms can also be of great importance in resource management. A number of funding mechanisms should be pursued in order to support the financial needs of healthcare services; these include grants for public–private partnerships (PPPs), and micro-insurance structures. Other strategies include, establishing cost-sharing mechanisms through which community members can afford to contribute towards the healthcare costs in a more manageable manner so as to ensure the sustainability of the healthcare programs.^[52]^ Some of the effective financial management practices include: budgeting, financial monitoring, and accountability, which are very crucial in utilization of resources. These are the best ways of managing resources in the face of disruption as they provide room for change and versatility. The healthcare systems also need to be flexible to changes for instance, when there is high demand or when there are supply chain disruptions.^[35]^ As a result, creating plans in advance and designing protocols for the emergencies can become the key to the quick solving of arising problems. For instance, storing up on some basic necessities and identifying the other possible channels to receive the supplies can reduce the effects of disruptions. Last but not the least, transparency and communication are important to efficient and sustainable resource utilization.^[85]^ Maintaining good relations between the healthcare providers, the policy makers, and the public aid in setting correct expectations and proper cooperation. Communication of the current situation with regards to resources, problems, and solutions to deal with scarcity can help in gaining trust and cooperation from the stakeholders. Involving the community in decisions that affect the management of resources like through public forums or feedback mechanisms can also improve on the efficiency and sustainability of health care programs.^[9]^ Hence, understanding how to manage healthcare resources sustainably in the face of scarcity and disruption requires the identification of needs, resource allocation, effective resource use, capacity building, partnership, experimenting new sources of financing, flexibility, and accountability. Thus, by incorporating these strategies, the healthcare systems will be in a better position to tackle for the scarce resources and ensure that the services are continued to be delivered despite the tough economic times.^[10]^

### 2.16. Impact assessment of sustainable practices on healthcare delivery and outcomes

The examination of sustainable practice effects on healthcare delivery is the process of identifying how sustainability has been incorporated into the healthcare systems and how it affects different aspects of care and health results.^[5,6]^ This process is useful in understanding the extent of the impact of sustainable practices and their role in enhancing the healthcare systems most especially the regions that experience constraints in resources and frequent disruptions.^[9,10]^ First of all, it is necessary to identify several areas to evaluate the effects of sustainable practices in the sphere of healthcare. This is because one of the major aspects is the effectiveness and the dependability of the health care services.^[96]^ They have also come with efficient use of resources and lean practices that are known to free up more health care resources.^[94]^ For instance, in the inventory management and utilization of protocols, the can minimize waste, mitigate stock outs, and guarantee availability of medical supplies as and when required.^[63]^ This can thus increase the dependability of the healthcare system thus providing timely and correct treatment to the patients even in the developing countries with scarce resource. It is also important to examine the availability and standard of care in this other area. Furthermore, there are practices that enable sustainable solutions in regards to health care services like community health as well as building the capacities of the local community, which can help to improve the fairness of access to health care services.^[74]^ This paper discusses how through training health care workers and making a push to start community health programs, health care systems are able to extend their services to cover the socially disadvantaged population and offer services to them where they are likely to be found. This approach can lead to better quality of care as it focuses on particular needs of a local population and makes sure that care is appropriate for a given culture and meets community’s preferences.^[53]^ Also, measures that support local manufacturing of medical facilities and that support cost-sharing models can contribute to making the health care services cheaper and available to a larger population.^[56]^ The effects of sustainable measures on the health status of the population is another important factor that has to be considered. Best practices in healthcare that promote sustainability may result in better health standards as well as disease management, decreased incidences of complications, and increased customer satisfaction.^[46]^ For instance, good management of resources and enhanced health systems will lead to proper disease management and reduced incidence of diseases which are life threatening. Likewise, improved utilization of services in the form of outreach programs and mobile clinics can lead to better diagnosis and treatment and therefore better health outcomes and less suffering from diseases that are avoidable.^[74]^ In addition, sustainability may help to strengthen and champion the flexibility of the health systems. Some of the measures that can be used include: variability in the allocation of resources, planning for contingencies, and integrating other stakeholders in the healthcare system.^[87]^ The enhancement of such resilience ensures that there is continuity of health service delivery and provision of care in the face of disasters and other crises thus improving the health outcomes despite the calamities. One of the most important factors that should also be taken into consideration is the efficiency of sustainable management. Measuring the costs of integrating sustainability into the healthcare systems is a 2-fold assessment of the direct and indirect costs.^[115]^ Several actions that are associated with sustainable management of resources include: waste management, resource management, and sharing of costs which can greatly reduce the cost. These savings can be channeled back into the health care services thus improving on the ability of the health care system.^[103]^ Also, efficient management can help sustain the financial aspect of healthcare programs hence having enough funds to cater for future and future needs. Hence, the impact assessment also entails the assessment of the other social and environment impacts of sustainability. Implementing sustainable measures that focus on environmental health, for instance through waste management and non-usage of hazardous substances in the provision of healthcare can enhance environmental health and in the process improve on public health.^[10]^ Also, involving the people in health interventions as well as in making decisions can enhance social relations and enhance chances of improvement in health and boost community’s capacity to handle challenges.^[22]^ These effects refer to the assessment of the sustainable practices that affect the healthcare system and the patients’ health results in efficiency, access, quality of care, and health improvement.^[17]^ It also entails the evaluation of the robustness of the health systems and their flexibility, the costs, and the impacts on the society and the environment. In this way, the above-mentioned aspects have been analyzed in detail so that the stakeholders can get a clear understanding of how sustainable practices can be made effective in improving the quality of health care systems and results.^[52]^ Mental health and psychosocial support (MHPSS) in conflict-affected areas are one of the essential aspects of humanitarian assistance and recovery.^[64]^ In such environment, persons and groups experience psychological problems because of violence, forced migration and loss of homes, and other forms of trauma related to conflict. In order to meet these needs in a proper way, there is need to have a holistic and culturally acceptable approach that will include mental health care and other psychosocial support.^[44]^ The effects of conflict on mental health are enormous whereby individuals are likely to develop psychological problems like anxiety, depression, post-traumatic stress disorder (PTSD), and other stress-related conditions.^[52]^ Conflict destroys social order and stability, undermines people’s confidence and trust in each other, and results in lack of access to even the most basic needs. These factors worsen mental health and limit people’s ability to seek help. Thus, efficient MHPSS in conflict areas is the one that targets both, the individuals and the communities while working within the contexts of the conflict.^[85]^ There is one major component of delivering MHPSS in conflict-affected areas and that is availability of mental health services. This includes the provision of services for the development of mental health facilities, the education of health care personnel, and the integration of mental health into primary health care.^[52]^ Destruction of health care facilities in conflict areas and shortage of mental health care providers are some of the challenges which may hinder access to professional psychological help. This means that there is the need to invest in building the capacity of the CHWs, nurses, and other front line workers through providing them with basic mental health care and psychosocial support.^[32]^ Other than the clinical services, community based approach have also been found to be very helpful in management of mental health in conflict areas. Such strategies include working with the community outreach in order to offer psychosocial support through group activities, counseling services, and support groups.^[21]^ There are various ways through which community-based interventions can be implemented for example: providing a platform where people can be able to share their experiences and receive emotional support and also engaging in community activities that foster unity and togetherness.^[20]^ These programs can be more culturally appropriate and acceptable and thus increase the chances of people seeking help through engaging the community members in the delivery of the support. Some of the important interventions include providing care to certain groups of people who may require special attention, including children, sexual violence survivors, and persons with disabilities.^[68]^ For children, some of the interventions may include child friendly spaces that provide play and learning opportunities, counseling and support to enable the child recover from loss and trauma.^[63]^ The care required by the survivors of sexual violence includes medical care, legal assistance, and counseling services for the short term and the long term. In order to make support comprehensive and accessible for people with disabilities it is necessary to modify services and materials based on their needs.^[64]^

Another effective strategy is the integration of MHPSS into other more general humanitarian interventions.^[43]^ This entails integrating mental health into all areas of humanitarian response such as food aid, shelter, and education. In the same way, raising awareness of other staff in other sectors like food distribution or shelter management, on how to identify people with mental related issues and how to offer basic assistance may strengthen the overall humanitarian response and guarantee that mental health is also addressed systematically.^[76]^ In conflict-affected areas, capacity development and problem solving skills are some of the most important strategies of the sustainable psychosocial support. This entails working to build the capacity of communities and improve their capacity to cope with various challenges that may arise in the society in the processes of social, economic, and political integration.^[20]^ Programs that offer employment, skills training, and social support help individuals and their communities deal with present stressors and heal from traumatic events. It is therefore important to systematically assess the efficacy of MHPSS interventions in order to strengthen and modify such programs.^[63]^ Acquiring information regarding the incidence of mental health problems, the success rates of the interventions, and the patient satisfaction also makes it possible to find out the existing deficiencies in the services and plan for the future. Also, involving the communities in the assessment of the programs helps the programs to be in line with the needs and wants of the affected communities.^[43]^ In conclusion, meeting the mental health and psychosocial needs in conflict-affected populations cannot be a 1 size fits all approach as it entails both short term and chronic interventions. Through extending mental health services within the primary care, adopting the community-based approach, targeting the specific population, and incorporating mental health within wider humanitarian assistance, it is possible to contribute to the positive mental health of persons and communities in the conflict zones.^[115]^ The importance of building and enhancing resilience, promoting community engagement, and conducting ongoing assessment and modification of the MHPSS programs are crucial in order to achieve the desired results in these difficult settings.^[105]^

### 2.17. Addressing the psychosocial impact of conflict on both healthcare providers and patients

It is, therefore, very important to focus on the effects of conflict on the psychosocial well-being of healthcare workers and patients since they are also key players in the conflict areas.^[115]^ The consequences of conflict are not only the physical ones, they also have an impact on all participants of the healthcare system: patients, healthcare professionals, and other workers, and it is crucial to take it into consideration, in order to provide high quality, empathic care.^[110]^ Health care providers who are practicing in conflict areas are likely to experience the psychosocial effects in different ways. Violence and death of colleagues, as well as the pressure from the need to give aid in the conditions of war and trauma, cause stress among the providers.^[106]^ The demands being placed on the healthcare professionals to deliver care in a variety of circumstances including in the context of emergencies and life threatening situations and combined with the emotional aspects of the work such as seeing people suffer and die can result in the development of burnout, compassion fatigue, and PTSD.^[107]^ These mental health problems may limit the ability to meet the patients’ needs or even the providers’ own health. Therefore, it is crucial to develop measures which would cater to both the psychological as well as the practical aspects of the health care workers.^[108]^ Therefore, making mental health support services available for the healthcare employees is a significant measure.^[109]^ This may involve counseling, peer support groups, and stress management among others. Such measures as ensuring that providers are able to share their experiences and feelings can reduce their feeling of loneliness and stress.^[100]^ Furthermore, working on the training of resilience and coping skills might help the healthcare workers to have tools to help them cope with stress and burnout. This also means that there should be tangible assistance especially for the health care workers. Some of the measures include having enough sleep, creating safe working environment, and giving the needed logistical and or material support to minimize the physical and emotional stress of the healthcare workers.^[18]^ Other ways include: making sure that the staff is well rotated and that they are well empowered to have their break and or time off to rest as this also helps in reducing stress when giving care.^[14]^ For the patients, the psychosocial effect of conflict is also equally important. Such factors as violence, displacement, and the collapse of social organizations cause psychological problems such as anxiety, depression, PTSD, and grief. The trauma experienced by the patients can worsen their physical health conditions, slow down the healing process, and reduce the chances of the patients’ interacting with the health care services. In order to meet the psychosocial needs of patients, all the mental healthcare and psychosocial support should be combined with the medical treatment.^[25]^ This support should include counseling and psychotherapy, support groups, and community interventions that aim at helping the victims of trauma to heal and cope. This means that instead of treating mental health disorders separately from other diseases, it becomes a part of the primary health care and thus patients get holistic care that includes their physical and emotional state.^[23]^ Also, designing a positive atmosphere in the health care organizations does play a crucial role on the mental health of patients. Educating the health care providers on how to identify and manage patients in psychological distress, empathetic communication, and patient’s involvement in the care planning enhance the experience and prognosis of the patients.^[64]^ Teaching patients on how to manage stress and how to find ways of coping with stress may also be useful to those who have been affected by conflict. Thus, it is possible to state that families as well as communities are vital in tackling the psychosocial effects of conflict. Involving families and communities in the care process can add more value to the care of patients and to the health care givers.^[42]^ Programs that have a focus on social cohesion, peer-to-peer support and services that support people, especially families, in conflict-affected areas may be beneficial. Lastly, assessment of the interventions and modification of the same based on the feedback received from the healthcare providers and patients is critical in enhancing the effectiveness of the psychosocial support.^[64]^ Thus the data was collected on efficacy of psychosocial interventions, analyzed the needs and difficulties experienced by the healthcare workers and patients, and used this knowledge to further develop the programs in a way that they are meaningful, appropriate, and beneficial.^[54]^ Hence, in order to manage the effects of conflict on the psychological well-being of both the healthcare workers and patients, it is imperative that mental health services are offered, material help is provided and the environment is made supportive.^[85]^ Thus, the application of these strategies will allow health care systems to enhance the health of both the caregivers and the care receivers, thus contributing to improved delivery of health care services in conflict-ridden areas.^[66]^

### 2.18. Collaboration and partnerships for sustainable healthcare

#### 2.18.1. Role of international organizations, local governments, and NGOs in achieving SDG targets

The SDGs 1 and 2 being pursued by the international organizations, local governments and NGOs have different yet interdependent roles.^[85]^ It is essential for the 2 organizations to work together in order to respond to various challenges of the modern world and promote sustainable development. Global players for instance the United Nations and its other related organizations like the World Health Organization (WHO), the United Nations International Children’s Emergency Fund, and the United Nations Development Programme are central in setting the goals and guidelines for the SDGs.^[90]^ Some of the roles of these organizations include the provision of technical support, research, and data in policy and standards making 4. They have the responsibility of leading in the provision of international policies, raising of funds, and making sure that the international policies are implemented. For instance, the UN sets the standards of SDGs and assists countries in their implementation, tracks progress through the global reports, and generates cooperation between governments, business, and civil society.^[6]^ They also play an important role in policy shaping and the promotion of SDGs on the international level. Local governments are very vital at the implementation of SDGs at the grassroots level.^[7]^ It is the organizations that transform the goals set by the global community into specific policies and actions that could be implemented at the local level and which are aimed at meeting the needs of certain communities.^[8]^ Local governments have the task of ensuring provision of SDG targets in urban planning, social services, education, and health. They are responsible for fund raising, formulation of policies, and provision of services that affect the people in the community. For instance, local governments may put in place policies that address issues to do with water and sanitation (SDG 6) or develop infrastructure to support sustainable cities (SDG 11). They also have a responsibility of identifying and working with the communities, especially in the determination of their needs and the provision of support towards their development.^[8,9]^

Nongovernmental organizations are very important in the implementation of the international standards since they help in translating the goals set to the local level. These are the implementing actors and they operate on ground level and are involved with the concrete issues regarding the SDGs, offering services, lobbying and support in the spheres of education, health, and environment. NGOs are important in development interventions because they bring in the much-needed expertise, experience, and community-level knowledge and understanding.^[12,13]^ Many of them operate on the ground level and focus on the advocacy of rights and needs of the vulnerable groups, as well as on the delivery of the activities which are in synergy with the state and intergovernmental activities. For instance, NGOs can provide health care services, education, and support various environmental conservation measures. The partners’ versatility and capability to respond to the changing environment make them strategic in the realization of the SDG targets.^[14]^ Thus, international organizations, local governments, and NGOs can effectively and expand the implementation of sustainable development. Such organizations collaborate with the local governments and NGOs in the development of the programs and their implementation to ensure that they meet the local context as well as the international standards.^[17]^ This partnership allows for maximizing resources, knowledge, and prevent replication and wastage thus ensuring the best outcomes are achieved in the end. For instance, an international organization may offer financial assistance and expertise to an NGO to develop a project that may be in line with one of the SDG targets while the local governments may support and supervise the project to meet the needs of the targeted community.^[32]^ The roles of these actors are interrelated and the actions of each of them support the actions of the others. International organizations initiate and lead the way and offer a roadmap, national governments are the ones to put into action and modify such policies and programs, and NGOs are responsible for implementation as well as promotion of change. In combination, they present a holistic conceptual framework to enable achievement of the SDG goals while taking into consideration the universal as well as the local.^[72]^ This helps in making sure that the efforts being made are well coordinated, proactive and involve all the stakeholders who are involved in the different communities across the globe. Hence, it is clear that the international organizations, local governments, and NGOs have their specific part in attaining the SDG targets.^[71]^ While international organizations set the direction for the world and coordinate the efforts on the global level, local governments’ institutions are responsible for the execution of policies and programs at the grassroots level, and NGOs focus on specific services as well as on lobbying for the rights of the concerned populations.^[74]^ They work hand in hand in the fight towards the achievement of sustainable development and the goals of SDG 2 and SDG 3.

#### 2.18.2. Case studies of successful partnerships and collaborative efforts in conflict zones

These positive partnerships and collaborations in conflict prone areas may involve a number of actors in order to tackle various problems and achieve meaningful results.^[101]^ Such partnerships involve the international organizations, local governments, NGOs, and community based organizations and groups. There is the possibility of achieving the intended results provided that their effort interrelate in a positive manner even in the most difficult conditions.^[100]^ There is a good example of the successful joint effort in the conflict zone: the reaction to the Syrian refugee crisis. When millions of Syrians became refugees due to the on-going war, several international organizations including the United Nations High Commissioner for Refugees and the World Food Programme in collaboration with local governments and NGOs offered important assistance. The United Nations High Commissioner for Refugees oversaw the provision of life essentials, such as food, shelter, and medical care.^[102]^ Some of the NGOs that played the role of direct care providers include Médecins Sans Frontières and International Rescue Committee that provided medical care, psychosocial assistance, and education. Some of the measures used by local governments in receiving countries such as Jordan and Lebanon include provision of services and infrastructure to the refugees.^[103]^ It enabled the refugees to get all round assistance which would meet their both short and long term requirements. For instance, the coordinated effort between the international organizations, who include NGOs and the local institutions in Somalia is an apt example of the centrality of partnership in the provision of disaster assistance in complex crises.^[104]^ In the times of armed struggle and humanitarian disasters, the United Nations Office for the Coordination of Humanitarian Affairs, and other similar bodies collaborated with Somalian NGOs and other local community organizations for the delivery of humanitarian aid. This partnership was very important in the provision of assistance in situations where there was the limitation of conventional supply chain. As a result of local knowledge and networks, these partnerships made sure that the assistance got to the people in need especially in the hard-to-reach and volatile areas.^[105]^ The role of the organizations also played a crucial role in building the trust as well as ensuring that the community participated in the program since that was key to the success of the aid. The next example of the successful collaboration in the context of the conflict-affected region is the response to the Ebola outbreak in West Africa in 2014 to 2016. The outbreak affected countries like Sierra Leone, Liberia, and Guinea among others, which called for international intervention.^[106]^ WHO with the help of other organizations such as Médecins Sans Frontières and Centers for Disease Control and Prevention, and other local organizations tried to combat the outbreak. It included treatment centers development, community mobilization, and launching of many health promotion activities.^[107]^ It also involved local governments who especially helped in the enforcement of the measures put in place as well as liaising with other countries. These efforts of all these diverse social actors were critical in preventing the spread of the virus and consequently, the elimination of the virus from the area.^[108]^ Due to the current war in Yemen, different humanitarian organizations have joined hands to help in the ongoing humanitarian crisis. The partners include the International Committee of the Red Cross, United Nations Agencies as well as local NGOs and they have been able to attend to the needs of the affected populace by providing emergency medical care, food, WASH in 120 cases. Such collaborations have been useful in the process of implementing programs in a region experiencing an ongoing conflict which is often characterized by limited access. These organizations have thus been able to provide much needed services and assistance to tens of millions of people affected by the conflict.^[111]^ There has also been engagement in advocacy so as to see to it that the humanitarian assistance continues to be provided and that the vulnerable groups are catered for. These case studies show some of the successful partnerships and it is important to note that collaboration in conflict-affected areas depends on the following. To achieve this, there is the need to ensure that there is good cooperation between the international organizations, the local governments, and the NGOs.^[113]^ Thus, each partner brings in its strengths and assets such as local knowledge, logistics, and technical know-how to the humanitarian mission and intervention.^[114]^ Besides, working with affected communities and gaining their trust is crucial in order to guarantee that assistance is provided in the most effective and appropriate manner possible.^[115]^ Positive lessons from collaboration in conflict-ridden areas depict that, it is possible to achieve results through partnership.^[116]^ Thus, international organizations, local governments and NGOs can avoid the lack of coordination, logistic and operative problems and, finally, make a significant difference in the lives of the conflict-affected populations.

#### 2.18.3. Ethical and policy considerations in conflict healthcare

This paper aims at identifying the ethical dilemmas that exist in the process of providing health care services and how they can be resolved while at the same time working towards achieving the SDGs.^[9]^ This is a delicate process especially so in the conflict areas and the high risk and low resources environments. There are many ethical issues in healthcare delivery; one of the important issues is the question of resource utilization.^[12]^ In situations where resources like drugs, medical supplies and equipments, and even personnel are scarce, decisions on who to attend to and who not becomes an ethical dilemma. This is further compounded in conflict areas where the levels of need are usually much higher than what is available.^[17]^ Healthcare providers and policy-makers are forced to make decisions on which interventions or patients should be given priority and this results in moral dilemmas and ethical issues. This is where the principle of SDG has to be considered while addressing the above-mentioned dilemma, with the principles of equity, justice, and universal health coverage in mind.^[18]^ SDG 3 that is centered on the health and wellbeing of all people especially through ensuring equal access to healthcare and provision of health services. In real life, this is done through the use of triage where patients are categorized according to the severity of their condition and the response given to them implies that everyone gets a certain form of care.^[19]^ Similarly, there are ethical values which are within universal ethical theories for instance, the utilitarianism that focuses on the value of the majority as the right thing to do is that which has the greatest value for the majority of the people; however, the ethicist must also consider the principles of equity and respect for individuals.^[15]^ There is another ethical problem related to the informed consent principle: it is difficult to obtain it in the conflict areas where patients are often stressed or not well informed. Some of the barriers include language barriers, patient’s poor understanding of the procedures to be taken, or through emergencies where there is need to act fast.^[2]^ In as much as health and well-being is part and parcel of the SDGs, patients’ autonomy, and consent must be considered within the context. This may entail breaking down information, resorting to representation, or else involving other members of the community to help explain.^[18]^ The principle of justice is also important in the consideration of health inequalities in conflict zones. SDG 10 that deals with reducing inequalities should be adopted when addressing inequalities in healthcare delivery and patients’ outcomes.^[37]^ In practice, this entails ensuring that every attempt is made to ensure that health services are extended to the hard to reach groups such as the refugees, the minorities and the people in the remote regions. Another issue is that when resources are provided for some groups and not others this creates ethical questions as to why some are neglected as much as others.^[15]^ Thus, the consideration of these factors entails a clear stand on the principles of equity and sound knowledge on the social factors that define health. Also, there is the problem of safety and security of patients and health care workers including the use of safe spaces for health care delivery in conflict areas. Violence is a concern when delivering health care services since there may be occasions when health care facilities and their users are targeted.^[17]^ A major challenge that has to do with the safety of those who work in the health sector as they seek to provide for their communities is maintaining healthcare service delivery within the context of SDG 16 that seeks to strengthen institutions, promote peace and justice. This involves creating and putting into place measures that can help to safeguard health care delivery systems and the personnel involved in this exercise, availing and promoting humanitarian assistance as well as compliance with the principles of the international humanitarian laws.^[74]^ It also entails ethical decision making when it comes to these dilemmas while at the same time taking into consideration the SDG commitments.^[85]^ Involvement of stakeholders and affected communities might help to make the right decisions with taking into account the views of all stakeholders. Ethical committees and advisory boards can help on decision-making in case of difficult scenarios and ensure that decisions made are ethical and in line with SDG principles. Some of the issues that fall under ethical dilemmas in the context of healthcare delivery include how best to tackle challenges of resources in relation to the SDG commitments, the issue of informed consent and justice as well as safety and security together with issues of accountability and transparency.^[75]^ Thus, ethical principles may be incorporated into decision making processes and practice so that health care organizations can respond to conflict situations and other contexts of limited resources and work towards SDGs.^[72]^

#### 2.18.4. Policy recommendations for enhancing the effectiveness and equity of health interventions

Improving on the delivery and distribution of health interventions is therefore a complex process that needs to tackle issues of system and implementation.^[12]^ Some of the policy recommendations that may foster the achievement of these objectives include: expanding equitable access to healthcare through cases of ensuring that the health facilities and materials are available to all populace especially the vulnerable groups.^[52]^ This includes the incorporation of measures that deal with social determinants of health which include education, water, and economic prosperity in an effort to the reduction of health inequalities. The effectiveness of health interventions may be enhanced by enhancing the health systems and the health workforce.^[63]^ This entails educating the health workers, improving the structures and ensuring that the required drugs and equipment are available. Moreso, the use of the technological tools like telemedicine and EHRs can improve service delivery and management.^[42]^ Policies should also pay attention to the issue of how health care should be linked up with other social and community support structures in order to work harmoniously and achieve the best results. Such measures that are based on community and its needs and circumstances may help develop more culturally sensitive solutions.^[74]^ Lastly, it is imperative that efforts be made towards ensuring that the governments, the international organizations, the NGOs, and the local communities work together to enhance the efficiency of the available resources. This means that through proper feedback mechanism, there should be transparency and accountability in making decisions and implementing health interventions in order to make the right decisions that will benefit the people.^[63]^

#### 2.18.5. Monitoring and evaluating health outcomes in conflict zones

Tracking and assessing health status in conflict settings is a systematic approach of assessing the effectiveness and effectiveness of the health care interventions and identifying gaps in the care delivery in the face of political instability and resource constraints.^[12]^ This process is important in order to guarantee that the health programs are efficient, fair, and relevant to the needs of the communities in need. First, the necessary targets and measures have to be defined in order to assess the health status including morbidity and mortality values as well as availability to health care. The following are some of the indicators that should be used in the conflict zones but they should be customized to the specific conflict situation.^[17]^ Some of the tools for data collection may include questionnaires, facility records, and observation but these tools have to be applied in accordance to the security and access situation in the area. Assessment consist of the analysis of the gathered information to determine the effectiveness and consequences of health interventions.^[64]^ This includes evaluation of the interventions in terms of the outcomes they are meant to produce, mapping out areas of weakness or poor performance, and appreciating the overall impact that the interventions are having on the health of the community. The implementation of the interventions should therefore be evaluated on a regular basis by both the providers and the consumers of health care services so as to determine their efficacy and appropriateness.^[86]^ The results of M&E should feed into modifications in health programs and policies for the better and to deal with the new challenges that may arise. It also entails fairness and equity; it also entails addressing inequities; and it also entails meeting the needs of the special and the vulnerable.^[32]^ In sum, it is possible to say that monitoring and evaluating health outcomes in conflict-affected populations has to be done with certain methodological compromises that are not always ideal but are rather realistic given the nature of the work in conflict-affected contexts. Thus it is important to ensure that health interventions are perfected and that resources are well deployed to enhance positive health in difficult situations.^[12]^

#### 2.18.6. Methods for assessing the impact of healthcare interventions on SDG progress

Tracking the effect of healthcare interventions on SDGs requires several approaches to guarantee that the interventions are efficient and produce the intended sustainable development impact.^[63]^ First of all, it is necessary to identify the concrete and quantifiable goals and objectives linked with certain SDGs. For example, if the aim is to track the progress towards SDG 3 (Good Health and Well-being), indicators could be the changes in the rate of maternal and child mortality, incidence of diseases or the proportion of population who have access to quality health care.^[85]^ These indicators assist in the assessment of the health interventions’ impact towards the achievement of the SDG goals. Data collection is one of the most important steps in the course of impact assessment. It can also entail the use of quantitative approaches including use of questionnaires, health facility documents, and statistical tools to assess the health situation and health care delivery systems.^[63]^ Quantitative data, including surveys, and semi-structured interviews, focus group discussions, observations, and document reviews can help to understand the implementation and impact of interventions in detail. These methods can explain the degree to which the interventions are appropriate for the local context and whether the affected population has access to them or not. Another method is the use of cross-sectional study with baseline and follow up.^[85]^ A baseline is set by collecting data prior to the initiation of an intervention with a view of using the data to measure impact. The follow up assessments are done at different intervals in order to check the changes that have occurred within the given period giving a clear picture on the effectiveness of the intervention.^[52]^ Such research designs as before and after intervention where data is collected from areas that have received the intervention and areas that have not received the intervention can also be used to rule out the effects of other variables. Outcomes are measured with the help of M&E frameworks which are very useful in determining the impact.^[36]^ These frameworks entail the development of structures for capturing the data, analyzing the data, and reporting the data. Some of the existing ones include the use of performance measures, timelines for assessment, and feedback mechanisms in order to ensure that results are employed to make the desired alterations to the intervention.^[43]^ Involvement of the stakeholders in the assessment process helps in the consideration of other people’s opinions. This include engaging the public, the health care workers, and the policy makers in assessment of the effects of the interventions. Their input can assist in determining the feasibility of the interventions, the applicability of the interventions and that the interventions are fair and suitable for the culture of the intended recipients.^[64]^ Statistical tools and techniques can also be used in the presentation of findings from an impact assessment, and this can be in form of data visualization and reporting tools. Dashboards, reports, and infographics can help explain the message to stakeholders such as the policymakers, funders, and even the public in a more simplified manner and help the audience to better understand the results and hence shape the future course of action.^[52]^ In conclusion, it is useful to note that the assessment of the role played by healthcare interventions in the realization of SDGs cannot be done in isolation, analytically or structurally, without the use of research tools, both qualitative and quantitative, involvement of stakeholders, and sound measurement frameworks.^[42]^ This way, healthcare interventions are not only efficient in delivering intended results, but also apply effectively to the objectives of sustainable development.^[85]^

#### 2.18.7. Frameworks for evaluating the sustainability and effectiveness of healthcare programs

Stakeholder frameworks for assessing the sustainability and success of healthcare programs are valuable resources to use in order to maximize the possibilities for program success and longevity. Both frameworks offer a different way of evaluating various aspects of program performance in a given community.^[52]^ The Logic Model framework is useful to depict the linkage between resources, activities, outputs, outcomes and impacts. Thus, outlining these elements, the Logic Model aids in defining a program’s theory of change, thus showing how resources and activities result in outcomes.^[63]^ This model allows the evaluators to have an organized way of measuring the effectiveness of the program, to be able to see if there are any missing links and to make changes to the program to make it more efficient if need be. The Theory of Change framework is a more detailed approach of how and why a particular program is anticipated to cause change.^[85]^ It involves setting the strategic objectives and identifying the condition and actions that have to be taken in order to accomplish these objectives. This framework facilitate to identify the assumptions and causal mechanisms that could determine whether the strategies of the program are likely to result to the intended outcomes and impacts of.^[86]^ It gives the overall picture of the program so that one can be able to look for any possible areas that may need some change. Results-based management (RBM) is more results oriented and emphasizes on the accomplishment of results and effects as opposed to performing tasks.^[21]^ This framework stress the need for goal setting focused goals and the use of performance indicators to report on achievement. RBM entails constant assessment of the program in order to determine whether or not it is accomplishing what it set out to do and whether it is producing the intended effects.^[35]^ It promotes an outcome-based management, guaranteeing that resources are well managed and that the achieved results are in accordance with the program’s goals. The Sustainability Assessment Framework focuses on the future sustainability of healthcare programs especially.^[85]^ Some of these include the financial health, structure, stakeholder involvement, and flexibility. This framework allows to determine the possibility of the program’s sustainability and effectiveness in the future, even when conditions change.^[63]^ It entails an appraisal of the program’s financial management, how resources have been mobilized for the implementation of the program and how the program fits into the existing health systems to make sure that the program will be sustainable.^[64]^ The Balanced Scorecard approach provides a comprehensive evaluation by measuring performance across multiple dimensions: financial, customer, internal process and learning, and growth.^[52]^ This framework assists in managing both the tactical and the strategic vision of an organization since it considers both the short-term and the long-term goals of the company. By checking on these various aspects, the Balanced Scorecard makes sure that healthcare programs not only accomplish their objectives but also do so in the most efficient manner while also being able to sustain their operations and encourage further development.^[74]^ Lastly, the Health Impact Assessment (HIA) assess the overall health consequence of a program or policy in place, whether positive or negative. This framework entails assessing, evaluating and preventing possible health impacts that may result from the program.^[65]^ HIA assists in guaranteeing that the interventions are not only beneficial to health but also tasked to minimize the impact of health disparities. Hence, these frameworks provide different ways of evaluating the efficacy of health care programs as well as their sustainability. The use of Logic Model and Theory of Change assists in identifying the program’s logic and pathways towards results while RBM emphasizes on outcomes and performance.^[63]^ The Sustainability Assessment Framework analyses the sustainability of the performance, while the Balanced Scorecard presents a general picture of the performance, and HIA is a tool which measures the health impacts. Altogether, these frameworks offer a sound method of checking on whether healthcare programs are on track and will continue to be so in the future.^[35]^

#### 2.18.8. Humanitarian logistics and supply chain management for conflict healthcare

Humanitarian logistics and supply chain management in conflict healthcare is the systematic and efficient management of resources so as to deliver medical aid and other related commodities and services to the needy populations in the most efficient manner possible.^[45]^ This step is important especially in war-torn areas where there are frequently no structures, access can be limited, and the problems are severe and manifold.^[47]^ There is always a need to plan and coordinate well to ensure that effective humanitarian logistics is achieved especially in the face of challenges brought about by unstable conditions.^[63]^ This entails mapping and controlling supply chain of essential medical commodities like drugs, vaccines, and equipments. This entails coming up with efficient transport channels, controlling the stock to avert stock out or stock in, and order the supply to be delivered to the correct place at the right time. This is because in conflict zones, there are other risks that have to be addressed by the logistics process such as the safety of transport personnel and assets.^[34]^ This may include persuading local authorities or militia for access, establishing security measures, and changing means of transport when the conventional ones are unavailable. Supply chain management in these contexts also includes the assimilation of local assets and knowledge.^[53]^ Engaging with local NGOs, youth groups and other community based organizations can be useful in the planning of logistics to increase the effectiveness of the interventions implemented as well as ensure that they meet the needs of the community.^[55]^ In addition, humanitarian logistics should be agile and flexible and should be able to respond to the challenges that may occur. This means that the organization should have a capability to engage in new conflicts or respond to natural disasters by having what is known as contingency plans and supply chain that can be easily adjusted depending on the situation that is at hand.^[64]^ In conclusion, it is submitted that efficient humanitarian logistics and supply chain management in conflict healthcare is a multifaceted process that entails strategic planning, security, collaboration with the local actors and flexibility in order to meet the needs of the affected population in conflict-affected areas.^[85]^

#### 2.18.9. Best practices for managing logistics and supply chains in unstable environments

Logistics and supply chain management in environment that is characterized by instability has to be handled with a number of guidelines that have been developed to help in dealing with issues such as unpredictability, lack of infrastructure and security threats.^[66]^ Some examples are: scheduling of adequate planning and coordination measures in order to prepare and counter calamities.^[54]^ This entails coming up with very elaborate logistics networks that map the risks that could arise and coming up with very robust strategies that will help in the face of a changing environment. It is therefore important to engage the locals, other organizations, leaders, and even global bodies in order to effectively work in chaotic surroundings.^[6]^ These partnerships can help in generating useful information, help in gaining access to the affected regions, and improve the functioning of the supply chain networks.^[10]^ This also allows the interventions to be culturally sensitive and therefore be in line with what the communities need. The need to put in place sound and secure means of transportation and storage of the solutions cannot be overemphasized 9. This entails employing several transport channel and mode to reduce the risk and guarantee the flow of supplies. Also, there should be provision for the safe storage of the medical supplies and equipment in order to prevent loss through theft or damage. This means that through assessing and reviewing logistics operations on the regular basis is that it makes it possible to identify and rectify the problems in advance.^[75]^ Real time data gathering and processing allows to make better decisions and to respond faster to changes that occur in the supply chain and require corresponding adjustments. Lastly, spending on training and management development of the logistics personnel guarantee that the teams are well equipped to handle the operations in difficult conditions.^[22]^ Skills that should be imparted during training include security measures to follow, measures to undertake in case of an attack; and management of resources. In the final analysis, these best practices can be rightly seen as critical in making logistics and supply chain management as agile as possible and resilient in any volatile and unpredictable operating environment.^[63]^

#### 2.18.10. Innovations in logistics and distribution to ensure continuous healthcare delivery

Logistics and distribution innovations are therefore vital in ensuring that health care is delivered in a continuous manner especially in difficult conditions.^[126]^ Technological developments in healthcare have been able to improve on the efficiency, effectiveness and geographical aspects of supply chains in the healthcare industry. One critical advancement is the availability of tracking and monitoring systems in real time.^[113]^ These systems incorporate global positioning system and radio-frequency identification technologies in order to give an up to date information concerning the position and the condition of medical items and facilities. It is beneficial for the healthcare providers as they can monitor the shipments, monitor their stock and in case of any problem they can attend to it immediately to ensure that supplies reach the right place at the right time.^[103]^ Drones, self-driving cars are the biggest revolution in the field of logistics. Drones are capable of delivering medicines, vaccines, and blood products in hard to reach or the war-torn regions where there is weak or no transport system.^[114]^ This technology cuts down on delivery time and avoids logistic constraints thus enabling delivery of supplies to needy communities within the shortest time possible. The application of autonomous vehicles such as self-driving trucks and delivery robots help minimize disruptions on the supply chain by automating transport and Daimler AG notes that human drivers add little value in unstable or hazardous conditions.^[26]^ Mobile health units and pop-up clinics are also other approaches that help in the provision of health care services to the target groups in the society. In addition, some of these mobile units are fitted with medical essentials, diagnostic equipment and, at times, personnel to attend to the sick on the field.^[64]^ They are especially valuable in regions which do not have permanent health care facilities or where these have been destroyed due to military action or disasters. These units are beneficial in the sense that they take healthcare to the affected populations thus closing gaps in service delivery and increasing access to care.^[85]^ Implementation of blockchain technology in the supply chain management of medical supplies has been made easy through the use of the below features. It provides an open distributed database to make records that cannot be altered and hence increase accountability and to curb fraud. It makes it easy to know the source of medical products and their handling thus minimizing on the chances of having fake or substandard products in the supply chain.^[66]^ There is growing application of data analysis and artificial intelligence in the supply chain and logistics management to enhance the supply chain management. Artificial intelligence algorithms use data to predict patterns of customer buying behavior, determine the most efficient routes for delivery and control stocks.^[68]^ These applications can predict whether there will be a shortage or surplus in the future thus allowing efficient supply chain management. This approach assists in decision making, optimal utilization of resources and reducing interferences. Lastly, the others that are packaging and cold chain management are also important in the delivery of healthcare through innovations.^[52]^ Adopting the advanced packaging techniques like the temperature sensitive packaging, critical health care products like vaccines and biologics are transported at the right temperature.^[74]^ This technology is useful in maintaining the effectiveness of the products and also secure distribution to the health care institutions. Altogether, all the described innovations contribute to increasing the efficiency and strengthening of logistics and distribution systems, so that healthcare remains continuous and adaptable to the needs of the affected populations.^[43]^

#### 2.18.11. Advocacy and awareness for conflict healthcare and SDGs

It is therefore important to take advocate and create awareness to ensure that conflict healthcare is in line with SDGs 1 and 2. Advocacy is the process of raising the awareness of the importance of attending to the health needs of conflict-affected populations and the immediate need for medical commodities, human resource, and other support services. As a result, through policies, IOs, and the public, advocacy can create policies, resources, and make sure that the healthcare in the conflict areas gets the recognition and support that it needs.^[53]^ It is also crucial to paint the picture of how conflict affects health outcomes and champions for strong and sustainable health systems. It involves raising awareness of the link between conflict healthcare and SDG, including SDG 3 (Good Health & Well-being) and SDG 16 (Peace, Justice & Strong Institutions) and to show how the improvement of healthcare can lead to other development goals.^[10]^ This is done through mass campaigns, public events, and civic and partnership with NGOs and community groups thus ensuring that there is increased participation and support.^[32]^ Thus, telling the stories of affected communities and describing the successful practices will help to mobilize people and make them more involved in the fight against healthcare problems in the conflict zones.^[20]^ In general, advocacy and awareness are the key factors that can help to mobilize the support and funding needed to strengthen health care in the context of conflict and ensure that such efforts are consistent with and contribute to the achievement of the SDGs.^[52]^

#### 2.18.12. Strategies for raising awareness and advocating for support for healthcare in conflict zones

This therefore implies that in order to bring awareness and to lobby for support for healthcare in conflict zones, a number of strategies must be employed in order to address various stakeholders and through various mediums in order to spread the message.^[32]^ At the core of these strategies is the development of the powerful messages that capture the essence of the health care needs in the conflict stricken areas.^[74]^ These narratives may include testimonials of the people who have been impacted by poor health care systems and the improvements that can be made through better support. Using newspapers, television and radio programs, and social media, and blogs, among others, the target population can be informed of the advocacy. Public awareness can be created and sympathy and support for conflict health care can be won through media campaigns.^[112]^ Continued engagement with journalists and media outlets for the provision of factual and timely information may also be useful in sustaining the attention on the issue. Other collaboration opportunities include NGOs, international organizations, and humanitarian organizations that can strengthen the advocacy work.^[70]^ Such organizations have an advantage of having well laid down channels and an already existing reputation that can be used to spread the message and mobilize resources. Joint activities, activities and materials can help to draw attention to health care problems in conflict areas and spur action.^[42]^ Another important approach is to involve the policy makers and government officials. The advocacy strategy should therefore involve direct lobbying, and policy briefs that call for more funds, policies and international cooperation. He added that in order to support their arguments, data and facts can be introduced during meetings, consultations, and public hearings and show how the sick and injured people in the conflict areas can be treated in compliance with the generally accepted humanitarian and development postulates.^[63]^ Awareness campaigns as well as educational programs and campaigns within target communities and the population are also important. Workshops, seminars and talks can also make people aware of the problems that exist in conflict areas and the need to support health care sectors in such regions.^[85]^ Awareness campaigns can assist in raising the level of people’s concern regarding the world and motivate them and organizations to contribute monetarily or through service and other means.^[32]^ The use of data and research to support the needs and effects of healthcare in conflict zone can improve advocacy.^[61]^ Thus, such materials as reports, infographics, and case studies can be considered as the most convincing when it comes to presenting stakeholders with statistics and success stories of proposed solutions.^[85]^ Hence, effective advocacy and awareness raising for health care in conflict-affected areas include developing sustainable and convincing stories, using media and partners, influencing the policy makers, informing the public and using evidence based information.^[43]^ All these initiatives help in building the support, resource mobilization and to ensure that there is proper provision for access to health care in conflict-affected areas.^[37]^

#### 2.18.13. Role of media and international bodies in highlighting the intersection of SDGS and conflict healthcare

International actors and the media also have a significant function in bringing attention to the SDG and conflict healthcare, each in its way, to promote change.^[74]^ The Media plays a very important role in passing information on the issues and requirements of healthcare in conflict areas. It is through news reports, documentaries, feature stories, and social media, the media can rally the world and show the effects of conflict on health of affected communities.^[52]^ Telling stories and presenting the stories of success and ineffectiveness of the humanitarian assistance, the media can influence the audience’s attitude and gain support for the healthcare agenda.^[63]^ Furthermore, media platforms can enhance advocacy activities by offering a platform through which experts, advocates, as well as, those who have been affected, can share their opinions.^[36]^ This coverage can show how meeting the health care needs in conflict-affected areas is in line with other SDGs, including SDG 3 and SDG 16. Through such reporting, the media is thus able to relate these problems to the general goals of global health and peace and the need to adopt holistic and sustainable strategies in conflict-affected health care.^[74]^ It is the International Bodies such as the United Nations, the WHO and other NGOs that define the dynamics of conflict healthcare and SDGs.^[75]^ These bodies offer statistics, undertake studies and set standards that define the international policy and practice. Their reports and statements can thus help raise awareness to the fact that the health care needs in conflict areas are related to and also affect the achievement of the global development goals where they provide evidence and policy guidelines and strategic directions.^[51]^ International organizations also help in coordination of the stakeholders, namely governments, humanitarian organizations and civil societies to address health care needs in conflict zones. It brings attention to how conflict healthcare can be linked to the SDGs by mobilizing resources, policy changes and capacity enhancement. Besides, through such partnerships, these organizations spearhead global campaigns and initiatives that link conflict healthcare to the SDGs, and promote awareness among the media, governments and citizens.^[18,74]^ Through their clout and experience, international organizations should be able to promote more cohesive and sustainable strategies to deliver health care services in conflict areas so that these initiatives remain relevant to the overall development process.^[129]^ Therefore, the media and international organizations are useful in raising awareness on how conflict health care is related to the SDGs. The media informs and influences public opinion and agenda, while international actors offer evidence, cooperation and advocacy for enhancing the provision of health care in conflict-affected areas and the achievement of SDGs.^[130]^

#### 2.18.14. Challenges to achieving SDGs in conflict-affected areas

The challenges of effective implementation of the SDGs especially in conflict-affected areas on health/well-being (SDG 3) and justice and institutions (SDG 16) include: technology, infrastructure, community participation/engagement and psychosocial support.^[131]^ However, the use of technology in the delivery of health care faces several barriers, such as poor communication network, scarce resources in technology, and vulnerability to technological risks.^[1]^ Infrastructure is critical in providing services that are basic and fundamental to society, but destruction of the same provides an initial threat.^[132]^ Healthcare facilities are attacked in military operations and this means that the provision of healthcare is limited as well as the availability of life-saving services.^[133]^ Other challenges include supply chain disruptions occasioned by road barriers, insecurity or even destruction of transportation infrastructure that hampers healthcare delivery.^[134]^ In countries like South Sudan, there are access barriers to healthcare in the form of no reachable roads and therefore stockouts of medicines, vaccines and surgical supplies. Inadequate durable and flexible infrastructure is also a problem in conflict areas.^[135]^ Tents and other temporary structures, or make-shift clinics, for instance, can be easily destroyed or made ineffective putting into focus SDG 3 which focuses on healthcare and SDG 16 on accountable and capable institutions.^[18]^ When such facilities are damaged or not functional, it hampers the health care needs of the people and hinder the development of interventions aimed at promoting peace and justice.^[131]^ COVID-19 has shown that community engagement is vital in the success and sustainability of humanitarian and community complexities in conflict areas.^[133]^ Nevertheless, there is a number of problems that can prevent the attainment of the SDGs, including: lack of trust between communities and authority, social dislodgement and disruption of social relations, cultural differences, and misconceptions regarding health needs.^[132]^ Exclusion of communities in decision making and healthcare service delivery is counterproductive to achieving SDG 3 and SDG 16 because health interventions may be rebuffed or wrongly implemented.^[133]^ This evidence is important for healthcare worker and populations in conflict zones as the psychosocial impact of conflict is far reaching.^[134]^ It is a known fact that conflict areas are more prone to mental health disorders such as PTSD, depression, and anxiety due to factors like trauma, death, and loss.^[133]^ Health care workers in the conflict areas may easily develop stress and burnout or secondary traumatic stress that diminishes their capacity to deliver quality healthcare.^[135]^

#### 2.18.15. Leveraging technology and health systems for SDGs in conflict-affected regions

To attain SDGs in settings affected by conflict, a full perspective of technology, health systems capacity, and psychosocial support will be needed.^[18]^ In regions where they do not have a good infrastructure, technology can improve healthcare delivery.^[1]^ To manage patient information in safe and efficient ways EHRs and Digital Health Systems can be introduced.^[133]^ But problems like unreliable internet and electricity in conflict zones can be solved with low cost, offline applications, such as local apps and mobile EHRs.^[134]^ Telemedicine and remote monitoring can be utilized to provide remote healthcare services such as consultations, diagnoses, and follow up care from a distance with patients coming to unsafe facilities.^[135]^ Technologies for satellite based communication can be used in rural or remote locations, while training local health workers in basic telemedicine techniques can bridge the technological skill gap.^[1]^ Additionally, healthcare delivery can be strengthened through strengthening health infrastructure, training and retaining healthcare local workforce, strengthening health system governance and addressing psychosocial needs in conflict zones, to promote peace and help meeting the SDG 3 and of SDG 16 respectively.^[135]^

#### 2.18.16. Strengthening M&E frameworks for effective healthcare interventions in conflict-affected areas

While M&E frameworks are critical in the implementation of SDGs in conflict-affected areas they are not fully developed.^[136]^ These frameworks make sure that the interventions are the right ones, the right way and at the right time.^[136]^ M&E strategies and tools include data collected from the healthcare facilities, mobile health technologies, telemedicine, and survey and interviews, real time data and dashboards, satellite communication monitoring and local health care worker training and support.^[137]^ Health outcomes assessment, process assessment, cost effectiveness assessment, community and stakeholder assessment, psychosocial assessment and peace and security assessment are some of the methods used in the assessment of healthcare interventions.^[136]^ Data sources include routine facility data, mobile health, telemedicine, surveys and interviews, real-time analytics and dashboards, satellite communication, and local healthcare worker training and support.^[137]^ Assessment of the healthcare interventions encompass health outcome assessment, process assessment, cost benefit assessment, patient’s perception assessment, psychosocial assessment, and peace and security assessment.^[137]^ There is need to have a strong M&E framework in order to assess the effect of interventions towards SDGs in conflict stricken areas. This on-going assessment leads to the realization of SDG 3 on health and well-being and SDG 16 on peace, governance, and stability in fragile settings.^[68]^ Through such frameworks, healthcare interventions can therefore be more effective, efficient and responsive to the challenges of conflict-affected environments.^[68]^

#### 2.18.17. The importance of the intersection between SDGs and resilient healthcare systems in conflict zones

Harmonization of the SDGs and resilient healthcare systems in conflict-affected areas will better position them to respond to the myriad of health needs.^[138]^ These are areas of violence, political instability, displacement, and lack of infrastructure, thus a collapse of health systems.^[139]^ To this end, the SDGs especially SDG 3 – Good Health and Wellbeing and SDG 16 – Peace, Justice and Strong Institutions should be embedded in resilient health systems that support recovery, stability and sustainable growth.^[140]^ Strengthening and rapid restoration of health services, development of sustainable health systems, crisis response, enhancement of health financing, and weaning off aid in resilient health systems.^[138]^ They also solve conflicts between the 2 parties, enhance management, and build unity in the society.^[139]^ SDG integration in healthcare interventions provides opportunities for timely monitoring and data informed decision making for efficient, efficient, and fair health interventions.^[140]^ This intersection is important for the improvement of the overall humanitarian condition of conflict-affected population.^[140]^

#### 2.18.18. Building resilient healthcare systems to overcome challenges in conflict zones and achieve SDGs

Healthcare system that is resilient refers to a health system that can quickly respond to the health needs that may arise in the course of a disaster and which can easily get back to normal in the event of disruption.^[141]^ It is developing through strong governance, efficient funding, qualified personnel, and outward-looking technologies such as telemedicine and mobile health applications.^[136]^ In conflict management, the health care systems must be strong enough to continue delivering services in the middle of conflict, displacement, and destruction of facilities.^[137]^ For these reasons, multifaceted issues, for instance, physical health status, mental health, social economic status and environmental factors cannot be addressed by a single discipline.^[142]^ Healthcare and the achievement of the SDGs are threatened by challenges such as violence, political instability, displacement, lack of infrastructure, scarce resources, economic instability, and mental health effects, spread of infectious diseases, barriers to health care, and effects on achievement of the SDGs.^[137]^ Both SDG 3 (Good Health and Well-being) hampers the attainment of universal health coverage and affects the provision of quality health care services, while SDG 16 (Peace, Justice, and Strong Institutions) affects the provision of quality governance structures and the rule of law in conflict-affected areas hampering the development of strong institutions that are necessary to support health system development in conflict contexts.^[142]^

#### 2.18.19. The impact of crises on healthcare systems

Crises such as those resulting from conflict, natural calamities or political instability are known to disrupt the healthcare systems and limit access to basic health care; reversing development gains on the SDGs.^[143]^ The loss of healthcare facilities, especially in weak or poorly endowed regions, is a big concern.^[144]^ For example, the Syrian Civil War has resulted in the loss of health facilities, medical facilities, and medical supplies which means that millions of people cannot get the medical care that they need.^[145]^ This has led to a tremendous regression in the achievement of SDG 3, which focuses on health and wellbeing for everyone at all ages.^[142]^ Crisis may also raise susceptibility to infectious diseases because standard hygiene standards are often neglected due to poor access to safe water and inadequate space, which could further stress health care systems.^[143]^ Mostly, conflicts lead to severe social and psychological problems, such as PTSD, depression, and anxiety, which are not considered in healthcare management.^[144]^ Inadequate ambulatory care, and particularly insufficient attention to mental health issues and crises, adds to the burden of disease.^[145]^ Displacement makes people to be living in camps or in other forms of shelters where they have no access to health facilities.^[142]^ This is because economic instability and shortage of resources lead to reduced government revenues and hence little funds to be used in the provision of social services such as healthcare.^[143]^ Crises in conflict zones complicate health sector problems by collapsing structures, overburdening the health care systems, rising incidence of diseases, and weakening governance.^[145]^

#### 2.18.20. Research gaps in the integration of SDGs into healthcare systems in conflict-affected regions

There remains a dearth of detailed conceptual and empirical literature on the integration of SDGs into healthcare systems in conflict-affected regions.^[136]^ There are several research gaps that can be identified, and all of them are connected with the interlinkages between SDG 3 and SDG 16.^[133]^ The present study identified gaps in the literature regarding the presence of specific comprehensive models for SGD integration in crises and limited research on the integration process within the health sector, particularly in Fragility, Conflict, and Violence environments.^[146]^ Sustainability and resilience are rarely considered; most works concern short-term reactions to healthcare challenges in conflict areas instead of strengthening health systems.^[133]^ Extending the analysis to include health, governance, and social determinants in conflicts areas is limited, this creates a gap in the relationship between institutions and health impacts.^[146]^ This article look at understanding the relationship between SDG 3 and SDG 16 in the context of healthcare delivery in conflict-affected areas through governance, transparency, and accountability.^[145]^ The topic of MHPSS in healthcare systems is seldom discussed, because conflicts are believed to cause psychological damage to the majority of population.^[136]^ Thus, the literature gaps discussed in this paper suggest that a more coordinated and durable strategy for SDG achievement in healthcare organizations, especially in conflict countries, is necessary.

#### 2.18.21. Healthcare needs in conflict-affected regions, ensuring long-term resilience and SDG achievement

This article explores healthcare needs and priorities in conflict-affected regions at the context of the SDGs focusing on SDG 3 (Good Health and Wellbeing) and SDG 16 (Peace, Justice, and Strong Institutions).^[136]^ Through interaction with various stakeholders, namely, local governments, international agencies, NGOs, healthcare providers and the affected populations, the study determined healthcare gaps and the most pressing healthcare needs within each context.^[133]^ The sustainability of health care strategies in conflicted affected areas is therefore essential considering the long term resilience of health systems is vital for the achievement of SDG targets even after the immediate conflict has ended.^[146]^ To ensure long-lasting impact and resilience, several key elements must be addressed: building local capacity: one way to reduce the dependency on external aid and make the healthcare system more self-sufficient is investment in the development and training of local healthcare personnel.^[136]^ Health care professionals need training in conflict sensitive health care practice and continuous education in providing culturally appropriate care in successive waves as the nature of conflict zones continue to evolve.^[133]^ Community-led health initiatives: participation of local community in the formulation, implementation and management of health services ensures that interventions are possible developed within the local context and more likely to be sustained through time.^[147]^ This method supports SDG 16 goals on governance and institutions by encouraging community ownership and participation.^[148]^ Integrating health systems: thinking systems fosters integration between health systems with other important sectors such as water, sanitation, education and governance.^[136]^ The sustainability of healthcare interventions is heightened when healthcare is aligned to development goals more broadly.^[133]^ Adapting to changing contexts: Health systems must be able to respond and change in the face of conditions changing, like disease outbreaks or new waves of displacement.^[136]^ This approach also grounds healthcare interventions based on both SDG 3 and SDG 16 in order to develop a more resilient healthcare system that will stand the test of time and react to crises in a timely manner.^[147,148]^

#### 2.18.22. Protection of healthcare workers and facilities under International Humanitarian Law

International Humanitarian Law (IHL) is the body of law in place which governs the conduct of war, with respect to civilian infrastructure such as healthcare systems being affected by armed conflict.^[149]^ However, being vulnerable, such as in conflict zones, combatants and their facilities are protected by and under IHL principles and treaties.^[150]^ These include the protection of medical personnel from attack, treatment of them regardless of their nationality and exemption from capture, detention or prosecution for their work in humanitarian areas during armed conflict.^[151]^ Under IHL Article 18 of the Fourth Geneva Convention, Additional Protocol I Article 12 and customary international law, healthcare establishments, such as a hospital, clinic and an ambulance are protected.^[152]^ They safeguard neutrality and impartiality so that healthcare workers can give care without being looked at as part of the conflict.^[149]^ Bearing the Red Cross or Red Crescent emblem shows respect for those emblems, which indicate that the person or vehicle so marked participates in medical or humanitarian activities and should not be attacked.^[150]^ Violations of IHL, including attacks on healthcare workers or facilities, can amount to war crimes, for which criminal accountability may be sought.^[151]^ Ensuring continued access to protection of healthcare infrastructure and personnel as well as those that need care involving the principles of humanity and neutrality, necessitates strengthening adherence to IHL, increasing accountability and mobilizing international support for healthcare workers in conflict zones.^[152]^

#### 2.18.23. Sustainable healthcare financing in conflict zones

One of the biggest challenges in making healthcare programs in conflict-affected regions sustainable in the long term is that burden of instability, shortage of resources and weak healthcare system contributes largely.^[153]^ Sustainable financing mechanisms are needed if health outcomes are to be equitable and stable. Community Based Health Financing (CBHF) models like Community Health Insurance pool local resources, community participation and solidarity to support sustainable pool of fund for health services.^[154]^ Healthcare systems can be stabilized with Public-Private Partnerships and services can continue despite challenging circumstances.^[102]^ Sourcing international aid from internationally donor countries and organizations has often become an important source of funding for healthcare in conflict zone often with unstructured funding that rarely generates long term impact.^[155]^ Conflict permit health insurance systems can be adjusted to enhance long term financial sustainably.^[154]^ Small affordable premiums paid into a micro health insurance scheme by individuals or communities gets them access to a predefined range of health services.^[102]^ In conflict-affected areas, governments must build up sustainable health care systems through better public financial management and higher domestic health budgets.^[155]^ These financial strategies apply to SDGs 3 and 16 to reach goals of better health and peace for future generations.^[155]^

#### 2.18.24. Innovative approaches to healthcare in conflict-affected regions

Conflict affected regions health care systems are troubled with instability, scarce resources and limited infrastructure.^[137]^ To tackle these issues innovative measures like CBHF, PPPs, International Aid and Donor Funding, Micro Health Insurance and Mobile Health (mHealth) Solutions and Government led Health System Strengthening, etc have been implemented.^[156]^ By aggregating local resources and creating feelings of ownership and accountability, CBHF have accessed more healthcare for vulnerable populations.^[136]^ Nonetheless, low income and low awareness of Community Health Insurance in some regions can result in low enrollment and a threat to the sustainability of the model.^[157]^ While PPPs have brought much needed efficiency in service delivery to states through the amalgamation of private sector efficiency with public sector equity targets, security and governance are the mainstays in attracting and sustaining private sector partners in conflict zones.^[156]^ International Aid and Donor Funding has been of utmost importance to give immediate relief and to set up temporary health infrastructure in Syria and South Sudan with conflict zones.^[136]^ But dependency on aid also brings us to unsustainable systems, and we need development of local health infrastructure in order to move from emergency relief to sustainable healthcare systems.^[157]^

#### 2.18.25. Cultural sensitivity in mental health and psychosocial support services in conflict zones

Given in a conflict-affected area, MHPSS services require cultural sensitivity.^[158]^ Respecting local belief and practices; developing trust between service providers and community members; addressing diverse trauma experience; and working in collaboration with local support systems.^[159]^ In other cultures, such as in African and Middle Eastern communities, mental health problems might be understood spiritually (see, for example, traditional healers or religious leaders) or communally.^[160]^ It is thus crucial that service providers build trust with community members, because people may already have incredible mistrust for external authorities.^[161]^ With regard to the collective cultural value of the community, group therapy or family based interventions may be more appropriate than individual therapy in post conflict settings.^[158]^ MHPSS interventions need to address the diverse forms of trauma experiences and be tailored to address the forms of trauma experienced by different groups.^[159]^ Indeed, good MHPSS intervention need effective communication which is hugely influenced by language – how people express distress and seek for help.^[160]^ Engagement and outcome are aided by adapting interventions to local languages and having counselors/mental health broadly familiar with the cultural context around communication styles.^[160]^ By working with local support systems, knowledge of the community is gained; the trusted support systems of the community are used to attract and cooperate with the community; and culturally appropriate interventions are ensured.^[161]^

#### 2.18.26. Assessing the impact of sustainable practices on healthcare delivery

Healthcare sustainable practices help maintain health services today and in the years to come. Different indicators are used to determine their impact on some critical areas, like access, quality, efficiency and patient satisfaction.^[162]^ These are indicators that tell us whether the healthcare system is improving and if it is operating in a sustainable way.^[163]^ Access indicators gauge how easily a population can receive health care, particularly the population at risk. Geographic access, financial access, equity in access, quality of care, efficiency of care, patient satisfaction, sustainability are the Key Indicators.^[164]^ Access indicators are indicators that tell us how far people are from healthcare services, particularly in remote or underserved areas.^[165]^ Quality indicators reflect how well health care services meet a standard and how well they improve health outcomes. Efficiency indicators are those measures expressing how efficiently are healthcare resources used for service delivery.^[161]^ A sustainable healthcare system is a healthcare system which makes maximum use of the resources which are available in the least wasteful way possible, and works in the least cost effective manner in the delivery of healthcare.^[162]^ Cost effectiveness, utilization rates, operational efficiency and resource allocation are the Key Indicators. Healthcare systems and their sustainability are dependent on the satisfaction of patients.^[163]^ Patient experience, patient centeredness, follow up and continuity of care and sustainability and long term impact are Key Indicators.^[164]^ An indicator of sustainability gauge whether a healthcare practice can provide longterm effects to benefit the population continuously.^[165]^ This includes financial sustainability, human resource sustainability and infrastructure development and maintenance as the Key Indicators.^[163]^ Financial sustainability answers the question of whether the healthcare system has a sustainable, self-sustaining financial base, while human resource sustainability refers to the ability of the healthcare system to have (1) the capacity to train, (2) retain, and (3) effectively deploy health workers.^[164]^ Infrastructure development and maintenance are a measure of the long term capacity of the healthcare infrastructure to cater for growing population needs.^[165]^ Lastly, indicators to gauge the impact of sustainable practices on healthcare delivery and outcomes are multi facets and offer an overview of what the healthcare system is doing.^[163]^ These indicators measure access, quality, efficiency, patient satisfaction, and sustainability to identify needed areas of improvement, to maximize usage of resources and promote long term success.^[164]^ Good performance on these indicators makes a healthcare system sustainable, and better able to provide equitable, good quality care in changing populations.^[165]^

#### 2.18.27. Ethical dilemmas in healthcare delivery in conflict zones

Based upon instability in war with limited resources, socio political dynamics of war healthcare delivery in conflict zones is a complex issue.^[166]^ Care principles collide with real world scarcity and security threats, which create ethical dilemmas in their provision.^[167]^ These dilemmas are illustrated by case studies, framed in terms of deontology and virtue ethics.^[168]^ In resource poor areas it has ethical implications regarding which patients get care, often to the neglect of others.^[169]^ Deontological ethics mean that healthcare providers have an obligation always to treat patients equally without regard to prognosis, and to utilitarian ethics which imply that providers may prioritize patients with a better prognosis for the greatest common good.^[170]^ However, it is difficult to balance these ethical frameworks in practice, since the duty to care clashes with the need to make the most of survival in resource limited settings. International aid organizations have to decide whether to compromise on neutrality, for example when operating in Yemen.^[171]^ Virtue ethics encourage giving on the basis of need in contexts of political malignity; deontological ethics ensure impartiality.^[167]^ Providers faced with balancing autonomy with the need to provide life-saving interventions are applying to emergency care.^[168]^ Healthcare providers are required, in deontological ethics, to obtain informed consent; when emergency situations make such a thing impossible, however, they must act without it.^[169]^ Treatments are administered based on the ethical character of the provider, yet argue no explicit consent is needed based on virtue ethics.^[170]^ Another challenge in conflict zones is the balancing of humanitarian aid versus combatant’s rights.^[171]^ Healthcare workers are to show justice and impartiality, and integrity in the way both civilians and combatants are treated.^[170]^ Not only do they need a theoretical understanding of moral philosophy but they also need a practical awareness of the problems of providing care in conflict.^[171]^

#### 2.18.28. Addressing the vulnerabilities of women, children, refugees, and people with disabilities in conflict zones

Specific groups in conflict-affected regions suffer heightened vulnerabilities based on the very particular challenges they are exposed to.^[172]^ Violence, displacement, and breakdown of social and health infrastructures frequently takes its heaviest toll on women, children, refugees, and persons with disabilities.^[173]^ With this understanding of the specific vulnerabilities of each of these different group, one can begin to design more targeted interventions and that humanitarian aid will reach those who are the most in need.^[174]^ Women in conflict zones face unique risk factors including sexual violence, reproductive health issues, economic disempowerment, psychological, educational disruptions, loss of livelihoods, discrimination and xenophobia, violence of all kinds, psychosocial challenges, physical and social exclusion, limited access to specialized services, and an increased vulnerability to exploitation.^[175]^ M&E of policy measures in conflict-affected areas play a key role in determining what can be improved, where equitable and effective interventions can be made, enhanced accountability, and adaptation to a changing context.^[176]^ Regular monitoring contributes to knowing how, and if, interventions do reach the right people, especially groups that tend to be left behind, and spotting potential unintended consequences or other damages of well-meant policies.^[169]^ Ensuring equitable and effective interventions is important for guaranteeing that all groups are receiving the needed care and protection.^[171]^ It permits for changes, and guarantees that everyone group is given care that it requires and care that its needs.^[172]^ A well-established principle in conflict zone interventions is the need for equity, which is achieved through monitoring to make sure that vulnerable groups who could easily be passed over in initial policy interventions, are included in policy adjustments.^[173]^ Transparent reporting and providing real time feedback mechanisms that enable stakeholders to track progress or address challenge at their right time, helps in holding accountable.^[174]^ In order to ensure that interventions are as effective while being culturally appropriate and in response to the real needs of those affected by conflict, community feedback is essential.^[175]^ Policymakers and aid organizations are able to continuously monitor changing circumstances and, in turn, adapt their strategies accordingly, ensuring their interventions remain relevant and effective throughout the conflict.^[176]^ Overall, it is not enough to simply provide humanitarian aid to the women, children, refugees, and people with disabilities in conflict zones; a multi layered solution is needed that requires immediate humanitarian aid as well as long term policy aimed at stabilizing, protecting, and restoring the lives of those whose vulnerabilities are routinely abused in conflict.^[175]^ This painstaking and ongoing process of monitoring and evaluating these interventions is equally important to understand their impact, equity, and ability to adapt as the need evolves.^[175]^ These groups in conflict zones can only be mitigated through such comprehensive efforts that foster resilience and long term recovery for the most vulnerable populations.^[176]^

#### 2.18.29. Ethical approaches to informed consent and addressing vulnerabilities in conflict-affected regions

The informed consent for medical procedures can be a challenge in the conflict afflicted and cultural and social logistical challenges. Some cultures are not accustomed to, or may even find it inappropriate, to secure individual consent, particularly in communal societies where decisions are made by family or community leaders.^[177]^ Healthcare providers are forced to balance respect for cultural norms with compliance to international ethical standards which favor individual autonomy.^[178]^ Alternative approaches to informed consent include community based consent, information materials that are simplified, and verbal consent in local language.^[179]^ Community based consent models allow us to work with the cultural value of a society while working with the medical care that the society deserves.^[180]^ When literacy rates are low and schools and hospitals have been compromised in areas of potential trauma, simplified information materials can communicate essential information in ways that those in the local population can understand.^[181]^ Even verbal consent in local language in clear and respectful way can also be an alternative.^[182]^ However, during armed conflict, violence, exploitation and neglect of certain sectors of the affected population, women, children, refugees, disabled people, is often a reality.^[183]^ Reproductive health needs of women in conflict zones are severely limited, and women in conflict zones are at high risks to sexual violence, exploitation, and discrimination.^[181]^ Health care providers need to be sensitive to such vulnerabilities and to ensure that culturally appropriate informed care processes take place, including around sexual and reproductive health.^[182]^ Children in conflict regions, too, face malnutrition, trauma and no access to healthcare, ones that may not be in a position to give a free consent unless they are old enough.^[183]^ Care must be delivered so as to minimize harm and trauma and sometime it requires consent from the caregivers or guardians on behalf of the patient.^[181]^ Displaced refugees in conflict-affected regions are unlikely to have access to consistent health care, and will be exposed to other diseases and other injuries. Efforts to obtain informed consent can be complicated by language barriers, fear of authorities and trauma.^[182]^ As conflict often disproportionately affects people with disabilities, the healthcare system needs to ensure that they do get the care they need and respect of their rights.^[180]^ For healthcare and humanitarian interventions in conflict zones to be effective, monitoring and evaluating policy measures is important.^[181]^ Interventions need to be equitable, responsive to vulnerable groups as needs change, adaptable as the situation evolves, apply regular monitoring to detect unintended consequences of aid delivery, and use community based feedback mechanisms to assess their impact.^[182]^ Humanitarian actors should think about the cultural context that informed consent is achieved in and discover other ways to do so, to ensure that interventions are equitable and culturally sensitive, and more powerful in the end.^[183]^

#### 2.18.30. Ethical challenges and strategic approaches to healthcare prioritization in conflict zones

There is frequently a lack of healthcare infrastructure in conflict zones, and so providers are forced to decide how best to use what resources they have available.^[184]^ Of age-dependent, severity of illness, and potential for recovery criteria, ethical concerns arise.^[185]^ Age-based prioritization targets children, the elderly, or pregnant women because of their high susceptibility and long-term outcomes from medical procedures.^[186]^ But this create equity issues particularly when working adults are left out in the consideration.^[187]^ Triage which involves sorting out patients based on the level of their need for care is also known as severity of illness prioritization.^[187]^ Yet the main idea of focusing on patients who are in the worst condition could be counterproductive because less acute but still dangerous health problems will not be treated at all, and this can have adverse effects on the patients who were ignored.^[184]^ Recovery prioritization capacity means identifying the patients who may recover without much interference and giving them a first chance at getting treatment.^[185]^ This approach raises some ethical questions, which are, patients who are considered to be near the death may be denied treatment even if they could have been saved by more time or different treatment in a less constrained health care system.^[186]^ Challenges and opportunities in enforcing prioritization in conflict zones include the following: constraint of resources, political instability, security threats, and cultural and religion barriers.^[187]^ Resource limitations result in the development of poorly integrated healthcare systems; security threats hinder the access of healthcare workers to the affected populations and provision of care in the order of the priority.^[187]^ Cultural and religious practices may influence the understanding of and adherence to prioritization strategies, which may prove difficult for international healthcare workers to manage appropriately and sustainably in a culturally sensitive manner.^[186]^ Potential areas for prioritization in conflict-affected areas include, cooperation between humanitarian actors, local government and international organizations, community participation, and use of technology such as telemedicine, mobile health applications, and drones.^[187]^ The challenges that hinder the achievement of health care policies call for the solution of the causes of conflict, the equitable distribution of resources, and the protection of the rights and interest of all stakeholders who are affected by conflict.^[187]^

#### 2.18.31. Monitoring, stakeholder engagement, and conflict resolution in conflict-affected healthcare

In conflict zones, the importance of monitoring and evaluating healthcare policy implementation cannot be overstated.^[142]^ It allows continuous evaluation and monitoring of effectiveness of interventions, filling gaps and ensures that resources are used optimally.^[188]^ Evaluation enables us to determine whether healthcare initiatives are being achieved amongst the most vulnerable populations and the outcomes they are intended to achieve.^[137]^ Organizations can enhance healthcare delivery, improve accountability, and respond to the changing context of conflict-affected areas by structuring feedback and adjusting strategies accordingly.^[136]^ This process makes sure that we intervene in ways that remain relevant and equitable and are able to address the healthcare needs that matter most.^[156]^ Equally important is that a wide array of stakeholders is involved in the decision making.^[189]^ Ineffective healthcare intervention cannot work without the high involvement of government officials, healthcare providers, the community field actors, and world bodies.^[188]^ Gathering these varied voices helps decision makers grasp what the community needs on the ground, what the specific cultural subtleties are, and what the practical challenges are likely to be.^[137]^ Inviting multiple health actors to participate in the design of an intervention is likely to enhance responsiveness to affected populations and increase the potential that interventions will be truly culturally sensitive, contextually sound, and receive buy in from the affected populations.^[136]^ Furthermore, it contributes to a sense of ownership among stakeholders to improve the sustainability and long term success of healthcare initiatives.^[156]^ Moreover, health care policy tends come about as the result of resolving conflicts and building consensus among stakeholders with disparate interests in its implementation.^[189]^ To be successful in solving conflict, there needs to be open dialogue between parties, a respect for differing viewpoints, and the accommodation of others’ opinions.^[136]^ Communication and collaboration among stakeholders can be facilitated to deal with power imbalances and competing priorities.^[156]^ Negotiation, mediation, and inclusive decision making are strategies that promote mutual understanding and a consensus building.^[156]^ In conflict zones, healthcare interventions are more likely to succeed and have a meaningful, lasting impact when stakeholders work in concert towards common goals.^[189]^

## 3. Conclusion

It is therefore important that the healthcare systems in conflict stricken regions incorporate the SDGs to help solve multiple problems. Some of the strategies include the use of telemedicine and mobile health clinics, building the health care systems and establishing community based health programs. The SDGs highlight the importance of strong health systems especially in conflict environment where there is little or no access to health care. In order to integrate the SDG goals into the humanitarian health programs, proper health needs assessment, facilities development and M&E should be done. There is also the need to advocate and create awareness on the SDG alignment in order to influence change and enhance the health policies and delivery systems. Technological advancement such as EHRs and mobile health application can be useful in improving the healthcare delivery in conflict areas. Community engagement is critical in the development of strong health care systems, conflict resolution, psychological support and general well-being. Strengthening healthcare through the means of rapid response teams and sustainable funding will also help to improve the availability and increase of healthcare services in conflict-prone environments. It is thus important to have good collaboration, proper use of resources and environmental friendly accommodations.

## Acknowledgments

We are grateful to Kampala International University Uganda for its support.

## Author contributions

**Conceptualization:** Chinyere N. Ugwu, Okechukwu Paul-Chima Ugwu, Michael Ben Okon.

**Investigation:** Chinyere N. Ugwu.

**Methodology:** Chinyere N. Ugwu, Okechukwu Paul-Chima Ugwu, Jovita Nnenna Ugwu, Fabian C. Ogenyi, Michael Ben Okon, Simeon Ikechukwu Egba.

**Supervision:** Okechukwu Paul-Chima Ugwu, Esther Ugo Alum, Val Hyginus Udoka Eze, Mariam Basajja, Regina Idu Ejemot-Nwadiaro, Simeon Ikechukwu Egba, Daniel Ejim Uti.

**Validation:** Chinyere N. Ugwu, Okechukwu Paul-Chima Ugwu, Esther Ugo Alum, Mariam Basajja, Jovita Nnenna Ugwu, Michael Ben Okon, Daniel Ejim Uti.

**Visualization:** Okechukwu Paul-Chima Ugwu, Val Hyginus Udoka Eze, Mariam Basajja, Jovita Nnenna Ugwu, Fabian C. Ogenyi, Regina Idu Ejemot-Nwadiaro.

**Writing – original draft:** Chinyere N. Ugwu, Okechukwu Paul-Chima Ugwu, Esther Ugo Alum, Val Hyginus Udoka Eze, Mariam Basajja, Jovita Nnenna Ugwu, Fabian C. Ogenyi, Regina Idu Ejemot-Nwadiaro, Michael Ben Okon, Simeon Ikechukwu Egba, Daniel Ejim Uti.

**Writing – review & editing:** Chinyere N. Ugwu, Okechukwu Paul-Chima Ugwu, Esther Ugo Alum, Val Hyginus Udoka Eze, Mariam Basajja, Jovita Nnenna Ugwu, Fabian C. Ogenyi, Regina Idu Ejemot-Nwadiaro, Simeon Ikechukwu Egba, Daniel Ejim Uti.
